# ECM Mechanics Control Jamming-to-Unjamming Transition of Cancer Cells

**DOI:** 10.3390/cells14130943

**Published:** 2025-06-20

**Authors:** Claudia Tanja Mierke

**Affiliations:** Faculty of Physics and Earth System Sciences, Peter Debye Institute of Soft Matter Physics, Biological Physics Division, Leipzig University, 04109 Leipzig, Germany; claudia.mierke@uni-leipzig.de

**Keywords:** cell and tissue mechanics, EMT, viscoelasticity, jamming-to-unjamming transition, extracellular matrix confinement, mechanobiology, individual and collective migration and invasion, stiffness

## Abstract

Cancer metastasis constitutes a multifactorial phenomenon that continues to confound therapeutic strategies. The biochemical signals governing motile phenotypes have been extensively characterized, but mechanobiological interactions have only recently been integrated into cancer cell motility models and remain less well elucidated. The identification of the biochemically and mechanically controlled epithelial–mesenchymal transition (EMT) of cancer cells, which occurs either completely or partially, has led to a major breakthrough and a universal phenomenon in cancers. In addition, a relatively new theory based on mechanobiological aspects called “jamming-to-unjamming transition” is being proposed to explain the transition of cancer cells to an invasive phenotype. The latter transition may help to better understand the different types of 3D migration and invasion of cancer cells. Similarly to EMT, the transition from jamming to unjamming seems to be controlled by molecular and physical factors, including cell mechanics and mechanical cues from the extracellular matrix (ECM) of the tumor microenvironment (TME). It is challenging to grasp the distinctions between the transition from jamming to unjamming and EMT, as they appear to be the same at first glance. However, upon closer examination, the two transitions are quite separate. Moreover, it is still unclear whether both transitions may act synergistically. This review highlights the most important breakthroughs in the transition from jamming to unjamming, with a focus on mechanobiology and extracellular environmental aspects, and it compares them with those of EMT. In addition, the impact of the TME, such as ECM scaffold and cancer-associated fibroblasts (CAFs) on the jamming-to-unjamming transition is discussed. Finally, the research frontiers and future directions in the field of mechanobiological research in cancer metastasis are outlined.

## 1. Introduction to Jamming-to-Unjamming Transition in Cancer

Solid tumors are intricate multicellular entities that can be regarded as ecosystems in which cancer cells establish heterotypic interactions with adjacent cells and their wider microenvironment [1,2,3,4,5]. These intricate crosstalk mechanisms of cells and their surroundings govern the rate of proliferation, the persistence of tumors, and the phenotypes of cell migration [1,4,5,6,7,8,9]. Cancer cell motility can be elicited through biochemical cues emanating from the microenvironment, involving growth factors, such as TGF-β, fibroblast growth factors (FGF), and hepatocyte growth factor (HGF), and cytokine release, which permit cancer cells to break away from the primary tumor [10,11,12]. Biochemical and structural cues affect the migratory behavior of cancer cells and govern whether they travel in a collective or individual migratory mode [11]. There is a growing body of experimental evidence demonstrating that the initiation of a collective multicellular movement and mechanical compliance in a tissue context is a process that is characterized by a phenomenon referred to as a cell jamming-to-unjamming transition [13,14,15,16,17,18].

From a biological viewpoint, there is still a lack of the mechanistic understanding of how, when, and why a cell embedded in a dense epithelial layer remains in place or, in contrast, undergoes cellular mobilization. In such cases, cell migration can occur cooperatively and collectively over long distances, for instance, in multicellular groups, swirls, stripes, and clusters, as in the transition from jamming to unjamming. These collective phenomena are critical for physiological processes like wound healing, embryogenic development, and morphogenesis, but also for the pathophysiology of carcinoma invasion into surrounding normal tissue [19,20,21]. From a traditional cell biology perspective, the cells undergo a reversible epithelial–mesenchymal transition (EMT), which can be either complete or partial/hybrid. Nevertheless, a holistic physical understanding of both transitions that can elucidate these collective cellular phenomena is yet to be developed and even not well explored in terms of the effect of the ECM microenvironment. It is not yet clarified how a confluent cell system can adapt jamming and unjamming behavior concurrently. It can be argued that this system has a unique topology, and each cell possesses the inherent ability to deform. A confluent cellular system maintains considerable internal degrees of freedom, such that cells can flow together and locally reorganize, thereby facilitating both jamming and unjamming dynamic behavior. There is notable recent experimental evidence for the idea that a confluent cellular collective may adopt both a jammed, solid-like phase and an unjammed, fluid-like phase, along with characteristic alterations in cell shape. In the well-known transition from liquid water to solid ice, there is a sudden molecular rearrangement from the amorphous disorder characteristic of a liquid to a far-reaching order characteristic of a crystalline solid. When particles aggregate to form a jam, such as in the jamming transition, there is no similar spontaneous structural order; disorder prevails in both liquid-like and solid-like phases, the latter being named a glass-like solid [22,23]. For these collective systems, the individual particles can engage with their immediate neighbors, and these mechanical engagements can branch throughout the entire system. The collective as a unit can then switch from a liquid-like and formable unjammed phase, which is appropriate for modeling tissue microstructure formation, like during branching morphogenesis, to a solid-like and frozen jammed phase, which is appropriate for sustaining the microstructure. Therefore, there is an urgent requirement for a more thorough comprehension of the evolution of metastases from the original cancer, starting with molecular and physical signals that trigger cancer cell migration. Cell jamming and unjamming phases have been linked to the formation of tissues in embryogenesis [16,24,25,26], wound healing processes, as well as the migration and invasion of cancer cells of certain cancer types (Table 1) [27,28]. The first evidence for the existence of cellular jamming and unjamming in a biological system has arisen from the traction force measurements within Madin–Darby Canine Kidney (MDCK) cells (strain II) by Trepat and coworkers performed in the progressing confluent epithelial monolayer in 2009 [29], and the presence of jamming in collective cellular systems has been unequivocally validated soon after [13]. In each individual case, traction fluctuations emerged in time periods exceeding the time it takes an entire cell to propagate by one cell length, which serves as further proof of the mechanical cooperative nature of the cells and the transmission of force on a scale beyond the individual cell. In this context, an exponential force profile, as described by Trepat and coworkers, has been identified as the hallmark of the force profiles that occur in jammed granular materials. The physics of jammed materials is still insufficiently explored, but it is assumed that the exponential character of the force profile results from the coupling of the following three common characteristics: tight packing, structural inhomogeneity, and long-range forces [30]. The mechanics of jammed granular material, by contrast, are determined through compressive stresses, while the mechanics of the cell layer are determined through tensile stresses [29]. Trepat and coworkers provided conclusive proof that the collective movement in a progressive epithelial cell layer is neither initiated by leading cells that pull the following cells along, nor by cells that move individually by themselves. On the contrary, every single cell, be it at the front margin or deep down in the center of the sheet, is involved in a complex power struggle that incorporates the localized energy production into a unified state of tensile stress. A mechanism of this kind is inherently holistic and therefore cannot be studied in an isolated cell. Such a mechanism would thus be very well suited to the migration and invasion of cancer cells during collective migration.

In this review, the principle of the jamming-to-unjamming transition is outlined and discussed in terms of understanding pathological scenarios like cancer cells in solid tumors. The focus of the review is on how mechanical stimuli like viscoelasticity or cell stiffness prompt cells to switch from a static state into a motile state, such as the transition from a blocked (jammed) to a free (unjammed) state. Various mechanical characteristics spread throughout and beyond these collectives and evoke these transitions. In contrast, it is debated how intrinsic factors, such as cell–cell interactions inside the solid tumor, and extrinsic factors, such as biomechanical characteristics of the ECM, like stiffness, favor the transition from jamming to unjamming and thus instigate cancer metastasis. Unraveling the physical aspects of TME involvement in cancer cell migration will shed light on the analogies and discrepancies between the transition from jamming to unjamming and the best-known transition from epithelial to mesenchymal cells, which governs deadly metastatic cancers. Finally, open questions in the research into jamming and unjamming behavior in the field of cancer research are highlighted.

## 2. Cell- and Tissue-Intrinsic Factors Contribute to Jamming-to-Unjamming Transition

**Figure 1:**
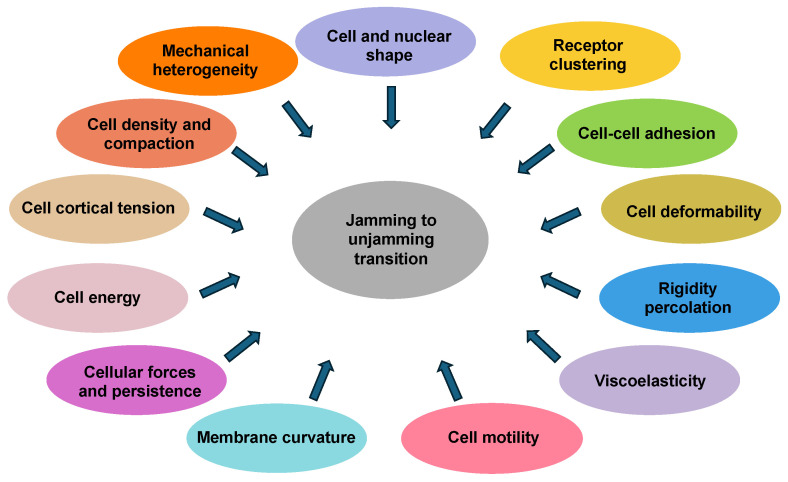
Cell- and tissue-intrinsic factors impact the jamming-to-unjamming transition. These factors include cell density, cell and nuclear shape, cell–cell adhesion, cell deformability, viscoelasticity of cells, cell motility, membrane curvature, cellular forces, and cell cortical tension.

The jamming transition in cellular systems is impacted by parameters such as cell and nuclear shape, cell density, cell deformability, cell cortical tension, cellular forces, cell–cell adhesion, membrane curvature, cell motility, and viscoelasticity (Figure 1). These factors do not necessarily appear to be equally important for regulating the transition from jamming to unjamming or vice versa. These elements are coupled to determine a preferred cell perimeter or target shape index, which dictates whether the collective response of cells is solid-like and jammed or fluid-like and unjammed. With rising cell density, the mutual interactions between the cells restrict their motility, which results in a deceleration of the whole system dynamics, which is a characteristic feature of the jamming transition.

Moreover, the solid-like jammed phase of the cells can be considered effectively “frozen” and is thus associated with tissue homeostasis, tissue stiffness, and mechanical stability. Conversely, the fluid-like, unjammed phase is actually “melted” and is hence endowed with the mechanical fluidity, plasticity, and deformability needed in the dynamic multicellular organization processes that shape the microstructure of the organ or promote the progression of cancer. How can a solid-like jammed phase arise within the epithelial sheet? When the layer has no voids or empty spaces, every cell is enclosed by its nearest neighbors. Therefore, every cell can be understood as being physically trapped by its neighbors. The strength of this type of confinement has a certain limit that can be specified in the guise of an effective energy barrier.

The energy barrier needs to be surmounted when the cell breaks out of its confinement and thus achieves cellular restructuring with its neighboring cells. The more solid the confinement, the higher the energy barrier. When this potential barrier becomes so large that it can only rarely be crossed, the cell will basically remain stuck in its place. In this trapped state, the cell will seldom break out of its cage and swap places with a direct neighbor. This leads to a structure known as a glass-like solid, which is assumed to be equivalent to any stable tissue in which epithelial cells are packed in a homeostatic manner [38]. Nevertheless, should the energy barrier in some way be surmountable, for instance by increased cellular propulsive forces, or should the energy barrier in some way be reduced or even eliminated, such as by the mechanisms outlined below, it would be easier for the cell to leave its confinement; in a multitude of such cellular collectives, the characteristic time and length scales for these cellular redistributions have been quantitatively assessed [13,14,22,39]. Occurrences like this would encourage cellular restructuring between neighboring cells, and finally, the collective as a unit could break out of the deadlock (unjam) and start flowing again [13,40,41]. This kind of unjamming could occur when a dense cell layer invades an unoccupied region to repair a wound, during embryonic development, as part of morphogenesis, or when cancer cells invade surrounding healthy tissue [42,43,44,45,46,47]. How jamming and unjamming of cells is associated with cell density is discussed in the following section.

### 2.1. Cell Density

Cells have to maneuver through tissues in pathological processes like cancer metastasis. The major question is how alterations in cell density affect the motile behavior of cancer cells in jammed tissue. Alterations in cell density have been shown to strongly control the behavior of jammed tissues throughout development. Enhanced cell density causes cell jamming, which is accompanied by cell volume alterations and can impact the fate of stem cells. When migrating as a collective inside confluent cell layers, cell layers exhibit fluid-like flow behavior, but on short time scales, they still act as a coherent and solid-like entity. The motion of a cell within these tissues is frequently severely limited by its neighbors that is referred to as cellular crowding [48,49], and consequently, it gives rise to glass-like dynamics. This solid-like behavior for short durations and collective flow for long durations is seen in numerous crowded particle systems that experience a transition from a supercooled liquid-like state into a glass-like state. Analogously, the collective movement of cells could be specified through a similar transition as follows: with increasing cell density, neighboring cells constrain the movement of each individual cell, thereby imposing collective motion on the cells. Individual cells, however, possess a high degree of deformability and self-generated movements; therefore, to comprehend the coexisting fluid-like and solid-like characteristics of a cell layer, it is indispensable to assess cell motility at both the multi-cell and single-cell levels. These liquid–solid transitions occur in densely packed tissues, where the interstitial space is absent and the packing fraction stays firmly fixed at unity [40].

The classical and frequently used model used to describe the jamming-to-unjamming transition is the Vertex model. In this model, the initial emergence of stiffness is determined by alterations in the characteristics of individual cells, like cell–cell adhesion, cortical tension, and volume compressibility, which offers an account for the liquid-to-solid transition observed in dense tissues. The following is a brief description of the model used to determine the transition from jamming to unjamming. 

The classical Vertex model for epithelia describes the apical surface of a tissue as a polygon tiling, where polygons stand for cells and the edges in-between for cell–cell connections. The mechanics are characterized by an energy that is calculated based on each cell’s deviation from a target area and a target perimeter. The target perimeter *p*_0_ has been found to control a phase transition from solid to liquid as follows: when the target perimeter value is low, the rearrangement faces an energy barrier, whereas when it is high, the cells can remodel themselves freely and the tissue is able to flow in the same way as a liquid. A common simplification is to model junctions with straight edges [50]. Over the past few years, shape-induced unjamming has been identified, with several newer publications highlighting shape-dependent unjamming in cancer clusters [51] and in intricate cancer-ECM microenvironments [52] and as far afield as asthma [31]. Consequently, the term cell density seems to be an inappropriate parameter, so the parameter cell shape will be discussed below.

### 2.2. Cell and Nuclear Shape

In biology, the cell shape is of primary fundamental relevance. Tissues are quite intricate and apart from alterations in cell shape, jamming-to-unjamming transitions owing to density variations induced by cell division, apoptosis, cell extrusion, and cell size alterations cannot be excluded. In addition, fluidization can also be caused due to the emergence of mobile topological defective sites. The average cell shape can affect key biological functions, like cell fate and the orientation of cell division. Due to the continuous cell division and apoptosis inside the cell layer, there is a strong disturbing impact on the shape and arrangement of the cells. These occurrences systematically cause the cellular orders to shift from a honeycombed packing to a polydisperse packing consisting of irregular polygons ranging from tetrahedral to nine-sided while favoring hexagonality [53,54,55]. In this context, Hertwig’s rule states that cell division orients itself along the longitudinal axis of the interphase cell [56], and it has been reasoned that this tendency promotes both stress relaxation and isotropic growth without the cells requiring to sense and transduce local mechanical cues [57]. Similarly, overcrowding of the airway epithelium resulting from proliferation and migratory events leads to Piezo1-facilitated extrusion of living cells from the monolayer [58]. Nevertheless, regardless of whether or not the effects are limited to the level of epithelial cells, in tissues that proliferate or die, it is observed that the polydispersity of cell shapes is the norm, and the regularly shaped honeycomb pattern represents the exceptional case. Due to these observations, cell shape has been proposed to be a factor relevant for the jamming-to-unjamming transition and vice versa [59]. The honeycomb structure has proven to be advantageous in terms of energy use for a cellular epithelial collection [60], because any division of the plane into cells of the same size results in a perimeter that is at least as large as that of a uniform hexagonal honeycomb [61]. Any other configurations that could be employed to subdivide the plane in cells of the same area, like triangles, squares, and parallelograms, would inevitably have a greater perimeter. Assuming that cell size determines energy expense, the honeycomb pattern provides optimal space utilization with minimal energy expense. In this arrangement, the tiling of the cell level is in regular order (i.e., in an orderly fashion). The ratio of the cell circumference to the square root of the cell area yields a dimensionless index of the cell shape, which is found to be close to 3.72 [18].

Cell shape has been explored as a biomarker for cellular fluidity within mechanically perturbed monolayers. In an earlier study by Ilina and coworkers, the classical Vertex model served to determine a critical shape index of 3.81 as the critical point for jamming transition [41]. Human bronchial epithelial cell experiments demonstrate that, independently of the amount of intracellular stress variations, cellular remodeling stops when the shape index is close to the point of jamming [13]. The application of compression stress on the MCF10A cells reduced the shape index, which converged to the critical threshold of 3.81, resulting in higher compactness of the cells. Conversely, the cell and nuclear shapes of the compressed 4T1 cells increased in length (higher shape index) and in variability when compared to the control cells. While the shape index relies on both elongation and tortuosity, the cell aspect ratio (AR) prioritizes elongation and attenuates tortuosity [62]. In 4T1 cells, a marked rise in AR coincided with a lesser rise in shape index, yielding elongated cells exhibiting straight edges. Hence, the cell elongation can more easily be assessed when the mean value of AR is plotted in relation to the standard deviation (s.d.) of AR. There is a positive linear relationship between the AR and the s.d. of AR, which agrees with [24]. The cell AR rose with the compressive stress, suggesting an unjamming effect, and their intercellular variability augmented. Compressed cells exhibited a tendency towards higher AR values and s.d. of AR. Enhanced cell elongation and shape fluctuations indicate a less ordered cell packing and a fluid-like phenotype and could be a predictor for elevated metastatic potency. These findings, along with the prior wound healing results, reveal a relationship between cell shape and the transitions between jamming and unjamming.

Apart from the cell shape, the nucleus shape has lately been associated with the tissue fluidity [63] and represents a decisive factor for the assessment of the tumor’s aggressive potential in the clinical classification of cancer [64]. In general, the nuclei of cancer cells are enlarged and softer compared to non-malignant nuclei [65,66,67]. Moreover, experiments on several cancer cell types, such as human breast cancer MDA-MB-231, human lung cancer A549, and human melanoma A375 cell lines, have revealed that the cells and their nuclei soften considerably during extravasation [68]. In concrete terms, this means that the spatial arrangement of chromatin, together with the localization of nuclear enzymes, is forced to alter as cancer cells squeeze through constrictions smaller than the diameter of the nucleus. Nuclear deformability has been shown to underlie a central function of cell migration in confined environments [69]. Thus, the next question is whether nuclear shape alterations correspond to cell shape alterations in cells exposed to mechanical constraints, such as compression. Since murine mammary carcinoma 4T1 cells expanded longitudinally under compression, a higher cell shape index coincided with an elevated nuclear shape index and high variability in nuclear shape, which has been linked to tumors with a more aggressive behavior [63].

These findings demonstrate that cell and nuclear shape indices rise with mounting compressive stress in unjamming transitions and act as key markers for cell migratory behavior and fluidity of tissues. Notably, mechanical compression led to elongation of the cell and nuclear shapes of metastasizing 4T1 cells, which experienced unjamming (Table 2), as opposed to stably jammed, non-tumorigenic MCF10A cells. The evidence shows that there is a nuclear jamming transition, in which the jamming of nuclei restricts cell motility beyond cellular jamming, with physical crosstalk between nuclei governing the resulting physical characteristics and tissue architectural organization [70]. In addition, Kim and coworkers used a computational model to identify nuclear volume fraction and nuclear anisotropy as critical factors in comprehending the evolving physical regime of the tissue. They found that a novel stiffness transition is coupled with a nuclear jamming, and consequently, Kim and coworkers proposed an integral function of the nucleus in controlling the emerging tissue mechanics and architectural organization [71].

Based on these correlation-based results, there is a general agreement that the unjamming is caused solely by alterations in the cell shape [72,73]. Thus, it can be questioned whether cells can control their movement through other crucial parameters governing the glass (thermal) or jamming transition (relies on geometric factors) [74,75]. Consequently, there seems to be a demand for synthetic model systems in which key features such as deformability, motile behavior, and cell density can be accurately governed. Along with closing the divide between simple models and intricate living systems, suitably conceived synthetic systems may be able to tease out the part played by cell shape in jamming and unjamming phases. Thus, a confluent synthetic cell monolayer system has been used to examine whether cell shape is linked to the transition from jamming to unjamming [76]. These confluent synthetic cell monolayers bear several weaknesses as they are an exaggerated simplification of densely packed epithelial tissues [76]. Different from natural epithelial tissues, there is obviously no adhesion between the cells, even though an effective activity-facilitated attraction is established. These “synthetic cells” can also be characterized by their fixed perimeter, a feature that is not present in living cells. The spatial restriction of the synthetic system is less pronounced compared to the density of natural epithelia; however, it prevails due to the presence of a dynamic internal skeletal structure. Nevertheless, the collective of synthetic cells exhibits several crucial features of living cells with regard to the jamming-to-unjamming transition [13,24,34,63]. Most remarkably, at the system level, cell shape fluctuation, often previously considered as noise, is found to co-scale with the average AR in a way exactly the same as seen in confluent epithelia, which confirms that they are suitable model systems [24].

### 2.3. Cell–Cell Adhesion

Cell–cell interactions are based on individual cell characteristics like adhesion and cortical tension. There is a glass transition of constant density, which is governed by the mechanical features of individual cells, including cell–cell adhesion and cortical tension (p_0_) and opposition to height variations (r) [40]. Confluent monolayers, in which mitosis (cell division) or apoptosis (cell death) are seldom, rely on the exchange between neighboring cells through processes termed intercalation [77,78] or T1 transitions [79]. This modification in cell behavior is not governed by cell density; however, instead it is regulated through the ripening of cell–cell and cell–substrate junctions [14]. In general, there are adhesive differences in breast cancer cells lines that are frequently used in exploring the transition from unjamming to jamming, such as MCF-7 and MCF-10A by Nnetu and coworkers (Table 1) [31]. Vertex and Voronoi models are used to characterize confluent tissue as polygonal cells and have revealed the key principles governing how this transition is orchestrated. These predominantly 2D models have demonstrated that cell jamming arises from the delicate balance between adhesion and cortical tension [40], active cell motility [41], and cell–cell orientation [15,80]. In a recent work, Kim and colleagues [81] proposed a novel 2D model that incorporates porous tissues and revealed that cell–cell adhesion is able to both unjam and jam tissues in a porosity-dependent manner. In confluent tissue, cell–cell adhesion promotes fluidization, whereas in porous tissue cell–cell adhesion it increases solidification. Most of these models concentrate on the 2D apical surface of epithelial cell layers. The shape alterations of cells and tissues that occur in morphogenesis or the metastasis of cancer are fundamentally 3D events. Smeets and coworkers solved this problem by devising a 3D deformable cell model (DCM) that precisely depicts the 3D shape of every individual cell and records the interplay between the cells by means of specific interaction forces [82,83,84]. DCM can naturally track alterations in cell shape from squamous to columnar epithelia, and it can characterize the performance of semi-confluent and fully confluent tissues. The phase transition of jamming has been investigated in an epithelial layer in terms of cell–cell adhesion and cell motile behavior. Using single-cell resolution to measure the dynamics of cell rearrangements, it has been revealed that cell motility fluidizes the tissue, and cell–cell adhesion fluidizes the tissue in the confluent phase, while favoring its stiffening in the porous tissue. Fluidization of confluent tissue has been observed to be concomitant with extrusion of cells.

Apart from mechanical markers, the cellular process of membrane transportation can impact the mechanisms of cellular and multicellular motility [85,86]. Disruption of endocytosis by modifying the levels of its key regulatory protein RAB5A [87,88] has been found to be enough to restore the motility of jammed epithelial monolayers. RAB5A induces large, anisotropic, and spatially related motility flows through a concerted increase in endosomal transport and macropinocytic internalization. These fluctuations affect the tension, topology, and dynamics of the proteins at the junctions and enable coherent cell movement across long distances. Thus, RAB5A can foster the elongation of aligned cell protrusions in conjunction with enhanced and more dynamic substrate traction, which together drives monolayer unjamming and collective motility in migratory cohorts (Table 2). The reawakening triggered by RAB5A has been identified as the outcome of a delicate crosstalk between alterations in cell adhesion and cortical tension, both changed in RAB5A monolayers due to the modification of endomembrane dynamics [15]. These events are supported by the efficient orientation of cell processes and dynamic traction forces to trigger directional migration of multicellular units. The motility restoration triggered by RAB5A is linked to an increasing length scale, which precludes an appreciation of monolayer dynamics based solely on an augmentation of local reorganizations [15]. RAB5A has been identified to orchestrate a number of distinct collective motility events in vitro and in vivo by reactivating the directed, concerted movement of jammed and kinetically stalled monolayers. RAB5A carries out this task by stimulating the generation of polarized, actin-based lamellipodia that produce traction forces that propagate over long distances efficiently via increased contact and stresses at the junctions. The enhanced mechanical connection also allows a cell to receive directional information coming from its neighbors, thereby forcing neighboring cells to orient their front–rear polarity, leading to a positive coupling between polarity and net displacement. In conjunction with elevated E-cadherin dynamics at the junctions to equalize the crosstalk between adjacent cells, volume, density, and strain variations, multicellular units can jointly assume a fluid-like behavior. These changes apparently result primarily from mechanical modifications due to overall disturbances in membrane transport. Nevertheless, considering the intimate connection between endocytosis and signal transduction, it cannot be completely ruled out that the amplification and rerouting of certain biochemical signaling routes, especially those originating from EGF receptors, underlie several of the modified mechanical characteristics. Significantly, these alterations in plasticity drive the motility of otherwise jammed and glassy monolayers, giving rise to invasive collective migration when physically restricted.

Cell–cell and cell–substrate adhesive forces are recognized to cooperate to generate a favorable cell shape [41,53,89]. Adhesions are important force-transmitting sites within cells and typically increase in strength as cells progress towards the site of jamming [14]. Cell–cell adhesion is conferred through cadherins, which are linked to the cytoskeleton [90], while integrin-driven cell–substrate adhesion is controlled through focal adhesions, which produce internal tension in the cytoskeleton [91]. In non-confluent tissues, reduced cell–cell adhesion leads to a decrease in cell compaction and cell–cell contacts, thereby enhancing tissue fluidity [92]. Cell–cell adhesion in confluent tissues is usually cell type-specific and varies according to the invasive capacity of the cells [93]. It has been demonstrated that strong cell–substrate adhesion combined with high traction helps to prevent confluent systems from becoming jammed [15,94]. Even small alterations in cell–cell and cell–substrate adhesion may have dramatic consequences for tissue mechanics and can be exploited to control cell retention [18,95]. How cell–cell and cell–substrate adhesion affects the jamming transition in a mechanically stressed monolayer has been analyzed for a specific example of breast cancer cells (see Section 3.1 below).

### 2.4. Cell Deformability and Energy

Each cell is viewed as projecting a preferred area—an area setpoint, A_0_—and deviations from this value are also viewed as spring-like energy losses. These losses were traditionally assigned to elastic deformations of the cell body (referred to as height elasticity), but they are currently viewed as encompassing alterations in active cell tension as well. Active cell tension can be maintained for long periods without viscoelastic relaxation [96] and can become more pronounced with rising cell density [97]. As far as can be ascertained, it has never been recognized before that the adhesion of the cell basis to the cell substrate has to be taken into account. For any cell inside the contiguous layer, the sum of all these contributions yields the total energy, which is simply stated in Equation (1) as follows:

(1) $$E=K_{A}{\left(A-A_{0}\right)}^{2}+K_{P}{\left(P-P_{0}\right)}^{2}$$

K_A_ and K_P_ denote the spring coefficients for fluctuations in area and perimeter, correspondingly, and A and P stand for the cell’s projected area and its perimeter, accordingly. The theoretical investigation of such systems revealed liquid–solid transitions together with characteristic alterations in cell shape [53,98]. Nevertheless, Bi and coworkers have been the first to carry out a detailed formal analysis that identified the occurrence of critical characteristic and a transition between liquid- and solid-like phases [13,40,41]. They determined three key characteristics, namely persistence, propulsion, and the favored cell shape p_0_ (adhesion/tension), which indicate the onset of such a transition.

- Preferred shape (p_0_): Factors that cause an elevation in cortical tension will result in a reduction of p_0_ and thus lead to a jamming of the system. Contradictorily, factors that lead to enhanced cell–cell adhesion cause an elevation of p_0_ and abolish the system’s jamming.
- Propulsion: Even random and non-correlated self-propulsion can produce forces strong enough to overcome energy barriers and unjam the congested layer. When the layer cells unjam in this manner, this occurs when the cells achieve a characteristic shape index of q = 3.81.
- Persistence: Self-propulsion forces can be even more effective in unjamming the layer when they continue for an extended period of time [99].

The first of these parameters, $p_{0}=P_{0}/\sqrt{A_{0}}$, is relevant for three principal reasons. The first place, p_0_, is dimensionless and can be regarded as a simple indicator of the preferred cell shape. In the second place, p_0_ represents a material characteristic of the cell, since it is determined through the relation between the cell–cell adhesion stress and the cell cortex tension [40,53]. In the third place, a distinct or threshold value of p_0_, denoted as $p_{0}^{*}$, exists, whose value is equal to 3.81. At this value, a phase transition occurs between the liquid-like phase and the solid-like phase of the cell collective [13,40,41] as stated in Equations (2)–(4) as follows:

(2) $$p_{0}>3.81\;fluid\text{-}like$$

(3) $$p_{0}=p_{0}^{*}\approx 3.81\;critical\;point,jamming\;transition$$

(4) $$p_{0}<3.81\;solid\text{-}like$$

The value of p_0_ can vary in each cell due to alterations in cell–cell adhesion, cortical tension, or a mixture of these and their many intrinsic regulators. Nonetheless, the theory predicts that the transition will be maintained simply if p_0_ is close to the approximate value $p_{0}^{*}$ ≈ 3.81. The preferred cell shape, p_0_, is compared to the actually achieved cell shape, as determined by microscopy, and it may deviate therefrom. The shape index characterizes the achieved cell shape $q=P/\sqrt{A}$. According to the theory of Bi and coworkers, the cell layer becomes solid-like and jammed when p_0_ < 3.81. Each cell is thus locked into a shape that diverges from its favored shape with q ≈ 3.81. When p_0_ > 3.81, the cell layer turns into a fluid-like, unjammed layer; hence, every cell can adopt its favorable shape and q ≈ p_0_. This means that the transition from solid to liquid takes place when the cells “wet” each other more strongly than they pull on each other, and vice versa.

The second parameter in the diagram of the jamming phase involves cellular propulsion. The propulsive forces generated by the cell layer on its underlying substrate alternate in space and time and are extremely concerted [22,29,100]. To close this conceptual void, the concept of a self-propelled Voronoi model was introduced and employed to propose a motility-driven unjamming transition [41]. This theory assumes that the propulsive forces can grow sufficiently powerful to surmount the energy barrier and thus cause the layer to unjam. Moreover, this model indicates that motility-related unjamming takes place when the observed shape factor q surpasses the identical critical threshold near 3.81.

The third variable relates to the constancy of the driving forces and how persistence intensifies those forces. Propulsive forces with a preference for constant direction produce a larger impact than those that are independent of direction. Collectively, this model characterizes the transition to jamming via three innate parameters, such as preferred cell shape (p_0_), propulsion, and persistence, and it orders these parameters in a phase diagram of jamming. The heterogeneity inside the cell layer probably leads to a shallower transition than the one predicted. Cells in a monolayer can be compared to an inert granule system as follows: when this system is on the verge of dynamic stall, slow particles are prone to aggregate with other slow particles and faster particles with faster ones, resulting in large-scale, highly correlated vortices, stripes, and clusters [23], referred to as dynamic heterogeneity [22,29,100]. Dynamic heterogeneity can arise spontaneously, even if the living cells show identical physical properties and the persistent structure is still amorphous and lacks correlation. In addition to dynamic heterogeneity, there are innate biological heterogeneities related to cellular variations in cell type, phenotype, size, adhesiveness, active motility, polarity, and cellular communication signals [14,101,102]. Even though these two types of heterogeneity, namely, dynamic heterogeneity and biological heterogeneity across cells, are separate, they can be mutually interactive and contingent. Thus, several questions arise as follows: Can biological heterogeneity obscure the phase transition and influence the dynamics of the transition to jamming in other crucial aspects? Conversely, can the dynamics of jamming be transmitted and reacted to through cells? How is the size of cell clusters regulated, and why do clusters form at all? More generally, could cell-to-cell heterogeneity in p_0_ and the consequent alterations in cell shape nonetheless serve as a structural hallmark to distinguish between migratory and resting/jammed phases?

The hypothesis that the cellular collective generally evolves toward an amorphous, solid-like, glassy phase, is supported by several scientists [14,22,23,31,39,100,103]. The mechanism or mechanisms whereby this solid-like, glassy phase arises is, by contrast, a matter of some dispute. Besides the cell jamming mechanism proposed above, other mechanisms, such as contact-based hindrance of motility [97], enhanced cell packing density [104], or, regardless of cell packing density, enhanced cell–cell friction stresses, as can be deduced from the ripening of the attachment bonds, have been hypothesized [14]. In general, the jamming transition takes place when the system experiences isotropic compression at a critical jamming density. Moreover, jamming has been identified as a result of shearing [105]. In addition, Garcia and coworkers proposed that frictional stresses are generated within the maturing cell layer on the scales of cell–cell adhesion and cell–substrate adhesion, and that these frictional stresses are linked to velocities in a way that defines the velocity correlation length [14]. Their theory is built around the idea of adhesion bond formation and breakage, but it does not take into account non-frictional stresses or the aforementioned rivalry of adhesion forces and cortical tension. Hence, a mechanism has been revealed that causes cell jamming and complements the mechanism highlighted earlier in this review. As a consequence, the predominant mechanisms of collective cell motility and the effects of cell jamming persist as unresolved questions.

### 2.5. Cell Compaction, Receptor Clustering, Mechanical Heterogeneity, and Rigidity Percolation

In 1955, it has been proposed for the first time that morphogenetic motility, comprising invagination, evagination, and layer propagation, may be partly due to variations in cell adhesion [106]. These morphogenetic movements could be described by simple physical models and may be universal in nature. Steinberg’s hypothesis of differential adhesion has been advanced to account for cell sorting in morphogenesis [107,108]. To consider the accompanying alterations in cell shape and even the cellular reorganization between neighboring cells during tissue reorganization without gap formation, Sulsky and coworkers have been the first to mathematically model the dynamics of the epithelial layer on the basis of a seamless and full tiling of the cell layer plane [109]. In this model, every polygonal cell is connected to its mutual neighbor–neighbor adhesion energies, that are represented with a mutual surface tension. The equilibrium is founded on the balancing of forces at every vertex, where cell–cell connections come together. In these Vertex models, the shape of the epithelium is depicted by a series of vertices that denote the intersection of three or more adjacent cells [110]. Therefore, these concepts are referred to as Vertex models. Sulsky and coworkers have also been the first to advocate a unified physical theory of epithelial mechanics, i.e., a theory that relates the physical forces inside the cell layer to the cell movements resulting from these forces [109]. In Vertex models, as mentioned before, every cell is portrayed as a polygon. The majority of Vertex models display either a cross-section of an epithelial layer or only the apical surface of an epithelial layer [110,111]. Even the experimental analysis by Ilina and coworkers is mostly based on 2D cross-sectional analysis by employing 2D trajectories (Table 1) [27]; however, for tissue sections, the analysis has been carried out using 3D trajectories for cell movements. In the field of tissue dynamics, 3D Vertex models have been developed [112]. The extension of classic 2D models to 3D systems necessitates the incorporation of parameters like mechanical polarization and topological transitions [112]. There is rarely an approach to model cancer progression, such as collective migration in 3D, for instance, the Merkel and Manning model [113]. Most transition models for (un)jamming are simplified and therefore consider cells as two-dimensional, which reduces the computational effort [111]. The positioning of the vertices and their connections has been used to generate a complete network of connections between the model cells. In addition to this skeleton of vertices and connecting edges, Vertex models contain equations of movement that determine how vertices change position based on the actual layout of the vertices. Moreover, numerous Vertex models incorporate guidelines that regulate alterations in the interconnections between vertices, thus enabling alterations in the adjacency relations between cells. These approximations are clearly appropriate in the context of densely packed cell layers, where the intercellular volume is insignificant, and are based on observational findings that cells in epithelial tissues are frequently organized in polygonal or polyhedral shapes. These cells are capable of moving to a certain extent independently of other cells [114]. For the dynamics of epithelia, such as the restructuring of connections, to be precisely characterized, cells need to be able to establish and disrupt bonds and need to avoid overlapping (with themselves). These are achieved through simple actions such as swapping adjacent cells (referred to as a T1 transition), and under specific circumstances, merging vertex/edges (referred to as a T3 transition). A T1 transition occurs when two vertices that have a short edge in common fuse into a single vertex, which then splits into two new vertices, causing the local network topology to become different [53]. In other words, a T1 transition event is when two adjacent cells drift apart while two of their nearest neighbors approach one another and become connected. The same applies to an event in which a link is deleted and a new link is created at the same location and perpendicular to the original link. Conversely, a T3 transition has been described in the context of the division of cells, where it leads to the nucleation of new cell junctions. An example of a T3 transition is when an interface between a vertex/edge is prevented by substituting the approaching vertex with two new vertices connected to the entity. The development of the system is therefore a mixture of relaxation until a mechanical equilibrium is attained and alterations in tissue connections in line with the specified cell reorganization processes. When delamination and/or apoptosis occurs, a T2 transition is performed, in which a cell shrinks to zero area and is removed [111]. A T2 transition takes place in extrusion/apoptosis phenomena, in which cells are removed from the monolayer in accordance with the disappearing connections. In particular, in T1 events, the total cell number within the monolayer is retained, which differs from T2 and T3 transitions.

T1 transitions have been found in both epithelial [115] and mesenchymal [116] tissues and are relevant in various phases of gastrulation and organogenesis [117] as well as throughout cancer metastasis [72]. Empirical evidence indicates that myosin II participates in the formation of tension at cell–cell junctions, thus governing T1 transitions within epithelial tissues [118,119]. Nevertheless, the interaction between mechanical and biochemical cues in these phenomena is still a matter of controversy [120]. In the Vertex model, every cell in the plane is considered as if it had a preferred value for its cell–cell contact extent—a perimeter target value P_0_—and deviations from this target value are regarded as spring-like energy losses [53,121]. As a rule, although not always [122], researchers have considered it helpful, perhaps even required, to divide this perimeter punishment into two competitive pieces [53,111,121]. In the first part, the contractile forces linked to the cell–cell junction work to reduce the perimeter of the cell–cell junction, and in the second part, the adhesion between the cells at their cell–cell junction works to enhance this perimeter. These impacts have contrasting signs and thus compete with each other. The overall impact on cell perimeter—contraction of the junctions opposed to adhesion of the junctions—can be summed up as total line tension, where it has either a positive or negative value. For instance, when contraction is more prevalent, the total line tension has a positive value, while when adhesion is more prevalent, the total line tension has a negative value [53,121].

There is a special case of activity facilitated unjamming reported by Sadhukhan and coworkers [123]. Activity-based unjamming transition within confluent cell systems is critical for embryogenesis, tissue injury repair, and the metastatic spread of cancers. The cells gradually alter their connecting characteristics, which are defined by an interaction variable *p*_0_, and start to move. What influence does activity have on the transition to unjamming? With the help of molecular dynamics simulations of the active Vertex model and analytical mode coupling theory (MCT), Sadhukhan and coworkers have demonstrated that the transition type stays like it is in steady state when activity is included [123]. The consistency of the simulation outcomes with the MCT projections indicates that the back-coupling mechanism between structure and dynamics governs the dynamics of relaxation. Beyond that, the first calculation of a dynamic length scale, *ξ*_*d*_, is introduced, and it is revealed that the increasing relaxation time is linked to a rise in the *ξ*_*d*_. In contrast to particulate glasses, the static length is linear with *ξ*_*d*_. These outcomes underscore the uniqueness of glassy dynamics in confluent structures and explain the prevailing experimental results.

### 2.6. Magnitude of Cellular Forces and Persistence Time for These Forces

Subsequent efforts to find a uniform explanation have been unsuccessful, partly due to the assumption that stresses exist within a cell layer, despite the fact that these have not been directly measured [100]. Cellular stresses inside a connected cell layer and how these stresses are spread out have been figured out through experiments, in which the amount of traction stress that each cell exerts on its substrate were determined [124]. Moreover, it has been explored how both normal stresses and tangential stresses spread out from each cell to its neighbors through their cell–cell connections [29,100,125,126,127]. So far, though, only some connections between cellular stress and cellular movement have been identified. When placed in a collective far away from any boundary, every cell within the collective has a tendency to travel along a path where the shear forces exerted on adjacent cells through mutual cell connections are kept to a minimum, which is a phenomenon referred to as plithotaxis. In contrast, cells near a cell-free gap have a tendency to apply a pulling force on their substrate that is directed toward the gap, which is a phenomenon referred to as kenotaxis [100,126,127].

Beyond the analysis of the forces of individual cells, the following discussion shows that confining particles to a short distance causes force transmission over a long distance. The slowdown in momentum in a granular system approaching jamming point has been considered at the system level. It is also meaningful to examine the dynamics at the microscale level. When the degrees of freedom in a system continue to decline, every cell can be confined to a small area of space, imprisoned by its neighboring cells with no chance of escapement. This trapping phenomenon arises when the interchange of locations between neighboring cells is restricted by an energy obstacle. When the self-propulsive force produced by a cell is not strong enough to surmount such an energy barrier, the cell is effectively locked in place. The consequences of such entanglements are far-reaching. The movements of a cell are hindered by its nearest neighbors, whose movements are hindered by their nearest neighbors, and so on. Mechanical forces are then passed on in waves that spread from cell to cell across the entire system, some of them over long ranges. This causes stationary clusters to expand and, beyond a critical threshold, the clusters become so huge that they cover the whole system. This eliminates internal degrees of freedom, so that cells are no more able to flow collectively or reorganize their locations relative to one another, and a finite portion of the system solidifies and hardens in a phenomenon referred to as “rigidity percolation” [128,129,130,131]. Therefore, rigidity percolation and jamming are regarded as two aspects of the same phenomenon [132,133,134]. In a disordered cell system, jamming inevitably leads to percolation of stiffness. The percolation of stiffness, in turn, is an integral characteristic of jamming. The living multicellular system is striking in that the percolation of stiffness in the developing zebrafish blastoderm [128] is very analogous to that in suspensions of loosely attractive inert colloidal particles [133]. In both living zebrafish blastoderm and non-living attractive colloid, particle–particle connectedness at the micrometer scale gradually alters as packing density moves closer to a critical value. In contrast, the overall stiffness of the system at the macrometer scale decreases so strongly that it can at times be approximated as a nearly non-continuous transition. In fact, it is now recognized that a large number of collective systems, comprising granular, colloidal, molecular, and cellular systems, display transitions from fluid-like to solid-like phases that are exclusively due to the percolation of stiffness and the accompanying kinetic blocking of jammed particles. The underlying idea is that, when it arises, jamming prevents the collective system from continuing to probe its immediate configuration landscape. In conjunction with percolation rigidity, cell populations are frequently heterogeneous throughout embryogenesis, with processes of cell sorting and cell mixing contributing to this phenomenon. Nevertheless, it remains an open question how cell sorting and mixing are impacted not merely by the classic concept of differential adhesion, but also by cell jamming/unjamming and the associated percolation of stiffness [45,103,121,135,136,137].

### 2.7. Viscoelasticity

Viscoelasticity has been largely excluded from the experimental analysis of the unjamming-to-jamming transition of cancer cells. As previously mentioned, there are several mutually dependent variables that affect the transitions of the jamming state, including, first, an enhancement of the packing density of the cells [39,104]; second, the cell–cell adhesion energy [14,40]; third, the strength of the cellular forces and the duration of these forces [14]; fourth, cell shape [14,18]; and fifth, the contact inhibition of locomotion [97]. The decrease in cell migration has an effect on viscoelasticity and therefore on the transition of the cell jamming state. The decrease in movement can be triggered by an elevation of the frequency in the vibration field or a reduction in the period of observation, by an elevation of the concentration of the system components and their rigidity, as well as by a lowering of the temperature, such as for the glass transition [138]. The density-dependent transition toward the jamming state in collective cell movement has been extensively examined [14,18,22,39,40,72]. Nevertheless, the associated modification of the viscoelasticity has not been assessed [138]. To close this knowledge gap, Pajic-Lijakovic and Milivojevic have come up with a systematic theoretical approach from a rheology perspective [138,139]. Angelini and coworkers [22] highlighted the density-dependent transition from convective to conductive mechanisms of collective cell migration. They showed that this transition affects the condition of viscoelasticity. Garcia and coworkers [14] examined the velocity correlation length with respect to cellular velocity. They showed that the correlation length rises with cell speeds below about 1 μm/min and declines with cell speeds above about 1 µm/min. The two cell movement tendencies are related to distinct states of viscoelasticity. A further rise in cell packing density reduces cell movement, which shows up as subdiffusive cell migration, causing the energy dissipation to be of anomalous character [39]. Consequently, there is broad agreement that density-induced cell reorganization can be divided into three regimes as follows: the convective regime, the conductive regime, and the damped-conductive regime, which takes into account the state of cell jamming. Each regime covers up to two states of viscoelasticity. Garcia and coworkers [14] demonstrated that cell monolayers exhibit amorphous solid characteristics at decreased cell speeds. For each regime, each viscoelastic state can be specified by a suitable stress–strain constitutive model that is consistent with the available experimental data. In line with these results, five states of viscoelasticity have been proposed by Pajic-Lijakovic and Milivojevic, such as the Maxwell model-based viscoelastic liquid of the convective regime, the Zener model-based viscoelastic solid of the convective regime, the Kelvin–Vogt-based viscoelastic solid of the conductive regime, the fractional constitutive model of the viscoelastic solid for the transient state of the damped conductive regime, and the fractional constitutive model of the viscoelastic solid for the cell jamming state of the damped conductive regime [138].

Consequently, besides cellular deformability, viscoelasticity seems to play a role in the collective movement of cancer cells. Viscoelasticity describes not the actual state of a system, but rather how a system accommodates itself to external or internal forces. This adaptation is achieved by a time-dependent mechanism that incorporates the storage and dissipation of elastic energy when the system’s structure alters [140,141]. The system behaves like a viscoelastic solid when the energy storage surpasses the energy dissipation. In contrast, the system acts similar to a viscoelastic fluid when the dissipation of energy rises beyond the energy storage. Storage aids in stiffening a soft matter system, while energy dissipation aids in softening. There are three key characteristics of viscoelastic fluids. The first characteristic is the failure of strain to relax under constant stress regimes. The second feature is the ability for strain rate relaxation occurring under specific circumstances. The third characteristic is the likelihood of stress–relaxation taking place under constant strain rate regimes. Conversely, viscoelastic solids behave quite differently. Main features of viscoelastic solids include the following: first, the capacity of the strain to relax when subjected to a constant stress; and second, the ability of the stress to relax when subjected to a constant strain. After the stress is removed, relaxation occurs and the residual stress returns to its equilibrium value. Viscoelastic solids can be categorized into those in which the residual stress is either purely elastic or a mixture of viscous and elastic behavior. In contrast, the residual stress experienced by a cell/particle in a linear viscoelastic fluid is fully dissipative and comprises purely viscous stress.

In the same system, different types of energy storage and dissipation can take place based on different load conditions. Remarkable instances include the reaction of a highly interconnected multicellular aggregate exposed to uniaxial compression from parallel plates [142,143] and the micropipette aspiration of cell aggregates [144]. The behavior of cell aggregates in uniaxial compression is characteristic of linear viscoelastic solids. This finding is consistent with the principle that stress at constant strain can undergo exponential relaxation, while strain at constant or zero compressive stress is capable of relaxation [142]. The accompanying stress relaxation time lasts several minutes and is related to the restructuring of cell–cell adhesion contacts and changes in cellular morphology [142,145]. Conversely, strain relaxation takes place through collective cell migration over longer periods of time, usually hours [142]. Micropipette aspiration focuses on a specific region of the multicellular aggregate, which causes cell–cell adhesion to break down and cells to migrate in the direction of the micropipette, thereby releasing a considerable amount of energy. These conditions prevent both cell strain and strain rate from relaxing, while stress at a constant strain rate can continue to experience exponential relaxation [144]. The performance of multicellular aggregates is similar to that of linear viscoelastic fluids. Nevertheless, this method for evaluating the viscoelastic characteristics of multicellular aggregates is inappropriate as a gauge of the viscoelastic characteristics of migrating cell collectives that are not subject to external forces. Cell migration is generally recognized to induce a strain that builds up over hours. This strain ultimately results in mechanical stress. The classifying of multicellular systems as viscoelastic solids or liquids is chiefly affected by the strength of cell–cell adhesion or cell cohesion, in which specialized membrane proteins like E-cadherin and integrins guarantee robust cell cohesion to epithelial and non-epithelial tissues. Epithelial cells establish robust E-cadherin-facilitated adhesion contacts that enable them to travel as cohesive cell aggregates that display viscoelastic solid characteristics [146]. Serra-Picamal and coworkers [42] and Notbohm and coworkers [101] investigated the collective migration of MDCK type II cells from packing densities that were equal to or lower than those of a confluent state, and found that the residual stress produced showed a correlation with the accompanying strain. In this situation, strain variations and residual stresses develop over hours, whereas stress relaxation can be observed within minutes. Pajic-Lijakovic and Milivojevic [139] came up with the idea of the viscoelasticity of migrating epithelial collectives that have a cell packing density less than or the same as the cell packing density in the confluent state. Collective cell migration leads to a progressive elevation in stretching extent over several hours. Each stretch results in a rise in mechanical stress and its relaxation within a bunch of short relaxation cycles at a constant stress per cycle. These consecutive cycles of stress relaxation led to the development of residual cell stress and its accumulation over the course of hours. Cell mechanical stress, cell packing density, cell speed, associated strain, and traction forces are unevenly distributed throughout cell monolayers [147,148]. An elevation in cell packing density, brought about by the build-up of mechanical stresses, hinders the relaxation of cell stress and alters the rheological characteristics of epithelial structures. The mesenchymal cells form weak cell–cell adhesions and move in streams [2,146,149]. The viscoelasticity of migrating mesenchymal cell clusters matches that of viscoelastic fluids [150].

The viscoelasticity and stiffness of cell monolayers, like the efficiency and persistence of collective cell migration, are heavily impacted by the viscoelastic characteristics and stiffness of the substrate templates. The majority of substrate templates, such as collagen frameworks, demonstrate viscoelastic characteristics [151], with collagen frameworks providing the primary structural support for several soft tissues. There has been a lot of work on how cell migration is linked to how stiff substrate matrices are, but the impact of collagen scaffolds viscoelasticity on cellular behavior is still largely unknown. Monolayers and substrate matrices that have direct contact with one another are subject to continuously recurring variations and are involved in intricate cause-and-effect cycles that affect the stiffness of the two systems. The link between these processes and their involvement in cell movement is a topic of ongoing discussion. The stiffness of cells and the substrate matrix are decisive variables that impact cellular reactions throughout development and in the context of disease. This stiffness is inextricably related to the viscoelastic characteristics of both the cells and the substrate that they interface with. In the subsequent work, the focus is on improving the knowledge of the viscoelasticity and stiffness of the microenvironment of tumors in the context of the transition from jamming to unjamming and the collective migration of cancer cells. To accomplish this, the factors of the TME that promote or hinder the transition from jamming to unjamming are discussed. There are essentially four objectives. The first objective is to characterize the primary physical parameters that drive stiffening in cell monolayers and ECMs. The second objective is to elucidate how tensile forces generated by cells promote the formation of stiffness gradients within the matrix. The third objective is to evaluate the influence of matrix stiffening on monolayer stiffness. The fourth objective is to assess how energy loss rates within the matrix affect the efficiency of cell migration.

## 3. Impact of the Tumor Microenvironment (TME) on the Jamming-to-Unjamming Transition

**Figure 2:**
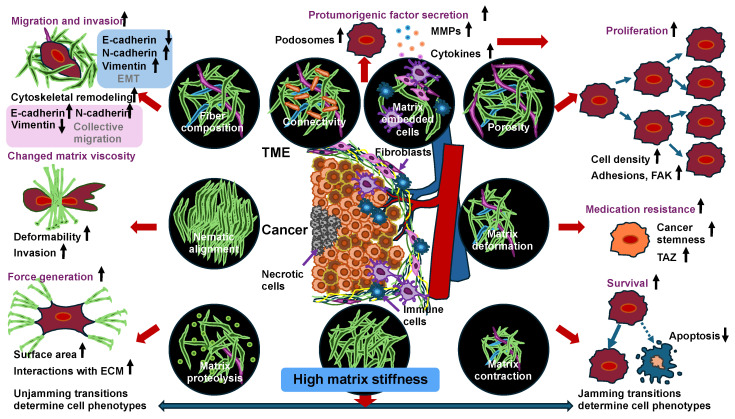
Unjamming and jamming transitions due to the elevated matrix stiffness of their environment. Adaptations of cancer cells arising from high matrix stiffness in primary cancer evolution can lead to jamming and unjamming transitions.

So far, the emphasis has been on the impact of cell–cell interactions on the jamming transition occurring in the invasion of cancer cells. Once cancer cells escape the primary tumor, however, their encounters with the surrounding environment, which is characterized by different biochemical and physical features, can drive metastatic progression. The TME has a complex structure. It consists of various cellular and extracellular constituents, like cancer cells, stromal cells, immune cells, cancer-associated fibroblasts (CAFs), and noncellular elements inside the ECM (Figure 2). All these elements can alter the mechanical signature of the entire tissue in the environment of solid cancers [2,5,152,153,154,155]. Besides biochemical cues, physical signals from the microenvironment can strongly influence cell proliferation, migration, and the potential for metastasis [153,156,157]. The capacity of cells to collectively undergo migration is impacted through microenvironmental factors like cell density and ECM characteristics. Due to elevated levels of ECM compounds like collagen and fibronectin, solid cancer tissues are frequently stiffer compared to normal healthy tissue [158]. Mechanical effects like ECM stiffness or interstitial pressure and mechanical forces such as tension and compression have been proven to modulate the metabolic activity, proliferative capacity, migratory behavior, and stemness of cancer cells [32,159,160] (Figure 2).

This process, whereby cells perceive and respond to the ECM, is termed cell–ECM mechanotransduction [3]. In 3D environments, (cancer) cells sense different levels of physical constrains in tissues and mechanical forces like compressive and shear stresses throughout individual and collective cell movement [161]. Elevated mechanical stresses in cancers can induce metabolic alteration in cancer cells, causing them to adopt stem cell-like characteristics, which promotes cancer growth and drives metastasis [162]. The mechanical characteristics of TME, such as solid stress, matrix stiffness, and interstitial fluid flow, alter during the course of tumor growth and evolution. The compressive stress within human cancers can range from 35 to 142 mmHg and influences cancer cells as well as the adjacent blood and lymph vasculature [162]. In addition to mechanical stress, the stiffness of the ECM and the physical constraints on cancer cells inside the microenvironment significantly contribute to the jamming transition. This poses several key questions as follows: How is the transition between jamming and unjamming initiated by a cell’s responsiveness to its mechanical surrounding? Is it possible that physical restrictions imposed by the stiffness of the ECM can “jam” cells and prevent them from metastasizing? Alternatively, are the mechanical characteristics of cancer cells capable of adjusting and moving more effortlessly through the stiff ECM, thereby facilitating unjamming? In the following subsection, the potential mechanisms by which cell–ECM contacts promote unjamming transitions will be discussed, and the possible involvement of mechanosensitive channels and transcription factors in facilitating the unjamming transition will be proposed.

### 3.1. Matrix Stiffness and Physical Constraints Govern Jamming-to-Unjamming Transitions

The cellular ECM is composed of an intricate scaffold of proteins and fibers, like collagen, elastin, and fibronectin, whose mechanical characteristics differ according to the kind of tissue [9,163]. Mechanosensitive proteins like integrins and focal adhesion kinases, such as vinculin and talin, play a critical part in turning mechanical cues from the cellular microenvironment into biochemical messages that drive cell movement [164,165,166]. Alterations in matrix stiffness have a particularly significant effect on cell activity and migration, which is conveyed via mechanotransduction paths and signaling processes. Specifically, breast cancer tissue has a noticeably higher stiffness of about 4 kPa compared to normal breast tissue of about 0.2 kPa, which highlights the importance of mechanical signals in the advancement of cancer [167,168]. Cells grown on stiffer substrates have been shown to develop an elongated shape, spread more widely, and move more quickly because of the enhanced contractile forces produced on stiff surfaces [169]. In environments with high matrix stiffness, such as 21 Pa, MDA-MB-231 breast cancer cells generate increased 3D traction forces, as measured by Afthinos and coworkers in soft, lithography-based, compliant microchannels, where the microchannels have been imprinted in a Polyacrylamide (PA) hydrogel with a stiffness in the range of 5 to 21 Pa with embedded nanospheres, referred to as hydrogel encapsulated microchannel arrays (HEMICA) [170]. The MDA-MB-231 cells form more robust cell–ECM adhesions and exhibit increased traction forces, resulting in faster and more effective migratory activity. In line with these findings, Beunk and coworkers revealed that the ECM in 3D environments significantly impacts the invasion of MV3 melanoma cells [36], with high ECM stiffness causing a jammed state by acting as a stiff roadblock that limits cell movement and low ECM stiffness making it easier for cells to relocate and migrate more freely, resulting in an unjammed state. Consequently, this finding has shown that mesenchymal-like cells can also exhibit a jamming transition, although there has been no determination of the specific shape index at their transition. From a biological point of view, these cells could only undergo a MET.

Primary solid cancers are surrounded by a basement membrane and are under increasing constraint as they grow, resulting in internal pressure accumulation induced in the cells due to proliferation [171,172]. The latest research by Cai and coworkers and Raghuraman and coworkers demonstrates how high pressure inside a spheroid can lead to spontaneous, explosive movement of cancer cells, which basically cancels out the cell jamming that is usually seen in the center of a spheroid [171,173]. This sudden movement, triggered by the pressure gradient between the spheroid and the broken-down ECM, enables the cancer cells to spread to distant sites. Specifically, burst-start migration has been defined as the abrupt, collective migration of cells out of a multicellular assemblage into the ambient matrix, similar to a “burst”. This phenomenon illustrates an unjamming transition in a 3D setting. Cell sorting inside 3D multicellular assemblies promotes heterogeneity and can lead to mechanical stress gradients and intercellular interactions that favor transitions from jammed and unjammed states and vice versa [174]. In the burst-like migration seen in a spheroid model consisting of epithelial and cancer cells [171], the cells are initially in a jammed state, which is recognized for being tightly packed and not very motile. In response to alterations in the boundaries imposed by the ambient matrix, the cells quickly transition to a condition of high motility, facilitating their collective penetration into the ECM scaffold. In this way, the cells break through the mechanical barriers evoked by ambient cells and the ECM scaffold that prevent them from migrating. Burst-like migratory behavior usually manifests itself in the formation of cell aggregates and not as individual cells, thereby raising the likelihood of micrometastases [173]. In this process, clusters of cancer cells migrate to remote sites throughout the organism, where they form small secondary tumors. Nevertheless, a stiff ECM is also capable of hindering or stall (jam) individual cells. When individual cancer cells migrate through the neighboring matrix, they are able to face restrictions like the dense ECM and physical barriers, which leads to impaired and less efficient movement. This obstacle can lead to the jamming of individual cells. In collective migration, by contrast, it is easier for the cells to unjam, which has been linked to effective metastasis.

The stiffening of the ECM is caused by the hyperactivity of proteins and enzymes, which results in the interconnection of collagen fibers and various other ECM constituents [175,176,177] and through the production of additional ECM proteins by cells [178,179,180,181,182]. In conjunction with the limited 3D migratory capacity of cancer cells, cells can dynamically reorganize and break down the adjacent matrix to generate migration tracks [183,184]. ECM stiffness and porosity affect the level of physical constraints that cells are exposed to. In reaction to varying degrees of restriction, cancer cells can alternate their mode of movement between mesenchymal and amoeboid motility [185,186]. Amoeboid migration is preferred when narrow spaces in dense tissues need to be traversed, while mesenchymal migration generally comes into play in more sparse surroundings. In addition, enhanced physical restriction due to high matrix stiffness can lead to the deformation of the cell nucleus, which in turn causes modifications in the polarity and gene expression of cancer cells [184].

The ECM is not simply a stiff, interconnected mesh of fibers, it also acts in a manner similar to a viscoelastic substance, which impacts the migrating behavior of cancer cells [160,163]. Beyond that, Bera and colleagues reported that the viscosity of the extracellular fluid can improve the motility of cancer cells through an elevation in the cortical actin meshwork density [187]. This change in cortical actin causes cell bulging and raises membrane tension, which boosts calcium influx and enhances RhoA-driven actin contractility [187]. Thus, myosin-dependent mobility and intracellular pressure rise, leading to a transition from unjamming. In addition, the plastically deformed ECM facilitates the restructuring of collagen fibers through cellular forces [188,189,190]. Cells can not only break down ECM scaffolds by secreting enzymes, but also physically rearrange them to move easier within the ECM [191,192,193,194]. In ECM scaffolds that are more plastic, cancer cells and stem cells can form microchannels inside the ECM to make it easier for them to move around, while more elastic ECMs are more likely to stop them from migrating. The mesenchymal stem cells stimulated a nuclear piston-based enlargement of the protrusions, which has been associated with the activation of mechanosensitive ion channels, leading to an elevation of osmotic pressure within the protrusions [195]. These results can be associated with the jamming transition, in which increased ECM plasticity facilitates the unjamming of cancer cells in a physical contextual setting. This demonstrates how cells can perceive and modify extracellular cues to enhance their locomotion across the ECM [163].

There are still questions about how the viscoelastic characteristics of cancer cells affect their capacity for sustained movement. When cells relax or break free from their blockage (unjam), they behave collectively like a viscoelastic fluid, while the jammed phenotype can be viewed as a viscoelastic solid. Atomic force microscopy and micropipette aspiration experiments have shown that individual cancer cells are easier to deform, behave more like liquids, and have lower apparent viscoelasticity compared to normal cells [141,196,197]. It has therefore been hypothesized that the reduced viscoelasticity of cancer cells, which is traced back to a reorganization of the actin cytoskeleton, leads to increased motility [197]. Another study describing the viscoelastic creep behavior of mouse fibroblasts and human embryo-spheroids also demonstrates that tissue relaxation after micropipette aspiration depends on deformation [198]. In addition, the relationship between the viscoelastic properties of cancer cells and the ECM and their mutual interference has yet to be comprehensively investigated. The role of viscoelasticity in the unjamming transition needs to be clarified, which requires improvements in tissue engineering [199,200,201].

**Figure 3:**
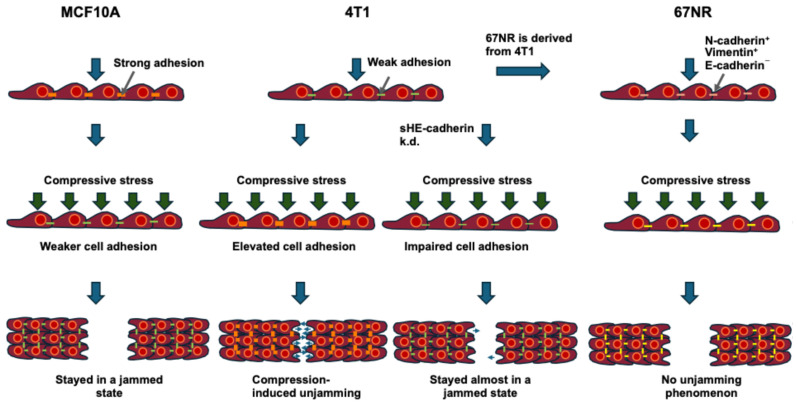
Schematic drawing illustrating the impact of compressive stress on the collective motility of MCF10A WT, 4T1 WT, and 4T1 E-cad KD cells, in which E-cadherin is knocked down and 67NR cells (derived from 4T1 and lacks E-cadherin). The orange lines indicate strong intercellular contact. The yellow lines display low intercellular adhesion lacking E-cadherin. The green lines point to low intercellular adhesion. The number of blue arrows corresponds to the relative cell migration speed in the process of wound healing or cancer progression. The green arrows indicate the compressive stress.

What impact has stress-like compression on the unjamming to jamming transition? Cai and coworkers have figured out how adhesion complexes help control the switch between jamming and unjamming of cells in tight monolayers when they are under mechanical pressure. When normal MCF10A breast epithelial cells and metastatic 4T1 breast cancer cells are exposed to pressure, the MCF10A cells stop moving (Figure 3), whereas the 4T1 cells take on a fluid-like appearance and move as a highly coordinated group.

During this process, the 4T1 cells lengthen and form strong cell–cell adhesions, while E-cadherin is destroyed at the cell–cell contact between MCF10A cells [32]. As mesenchymal hallmarks are not elevated in compressed 4T1 cells, this compression-based transition differs from EMT. Compression decreases tensile stresses in microstructured cell clusters and inside the monolayer for MCF10A and 4T1 cells. Consequently, Cai and coworkers have revealed that increased intercellular adhesion and decreased traction inside the cell layer control the different cellular responds to mechanical compression and promote jamming–unjamming transitions (Figure 3) [32].

To investigate the function of E-cadherin in more detail, Cai and colleagues weakened cell–cell adhesion through the knockdown of E-cadherin (encoded by the CDH1 gene) in 4T1 cells and examined the impact of the knockdown on compression-induced unjamming in comparison to cells that were transduced with scramble shRNA [32]. Increased E-cadherin levels in unjammed cells imply a cell–cell adhesion function in tight monolayers subjected to mechanical compression, and it is established that E-cadherin depletion switches the migration mode of cells from collective to single-cell movement [202]. The elimination of E-cadherin first caused cell motility to rise, which is in line with Ilina and coworkers [27]; however, collective movement in E-cadherin knockdown cells (E-cad KD) has been diminished under compression [32]. Exerting compressive stresses of 600 and 1200 Pa reduced the collective migration of E-Cad-KD cells, thereby slowing the rates of wound sealing from about 31% at 0 Pa to about 9% at 1200 Pa [32]. The increase in E-cadherin expression through compression is markedly decreased in E-Cad-KD cells compared to scramble cells. This upregulation could not be attributed to recovery of E-cadherin expression, since expression levels stayed suppressed over an 18 h time period in the absence of compression. In addition, the mesenchymal markers N-cadherin and vimentin were strongly expressed in KD cells and became even more elevated expressed when subject to compression. Vimentin can be blocked in E-cad KD cells to investigate the possible involvement of this mesenchymal biomarker during jamming–unjamming transitions. These outcomes demonstrate that E-cad KD cells stayed in a jammed state during compression, like the result observed in MCF10A cells [32]. To evaluate the general relevance of these findings, the impact of mechanical compression on the non-metastatic mouse breast cancer cell line 67NR has been investigated. The 67NR cell line originates from the same primary breast cancer source as 4T1 and expresses N-cadherin and vimentin, but it lacks E-cadherin expression [203]. Even though 67NR cells display enhanced cell locomotion, resulting from higher cell–substrate adhesion during compression [93], no unjamming phenomenon has been detected during the compression of 67NR cells. Based on these results, it can be proposed that the E-cadherin expression and its localization are necessary for compression-facilitated, fluid-like unjamming transitions. In line with the results for MCF10A, compressive stress could not raise E-cadherin levels with 67NR cells. Consequently, compressive stress markedly impaired the coordinated movement of 4T1 E-cad KD cells, and an upregulation of E-cadherin expression is needed for compression-triggered unjamming. Therefore, it can be hypothesized that cell–cell adhesion functions as an important regulatory and effective factor in compression (Figure 3) [32].

### 3.2. Impact of Fibroblasts in the TME, Such as Cancer-Associated Fibroblasts (CAFs), on the Unjamming Transition

So far, the emphasis has been primarily on unjamming transition, which is controlled through cell–cell connections, where the interactions between the solid cancer and cancer cells with the ECM have been largely ignored. There has been growing attention recently paid to the important role of the overall TME and how the interplay between various cell types promotes cancer advancement [4]. Fibroblasts are a prevalent cell type present in the TME and perform a critical function in tissue repair processes [204,205]. CAFs are of considerable importance in cancer biology because of their capacity to secrete soluble factors and reorganize the ECM, which imparts effects on cancer cell behavior, for instance, in connection with jamming transitions [206,207]. The restructuring of the ECM through CAFs alters the mechanical characteristics of the ECM and the subsequent forces to which cancer cells are exposed. CAFs generate collagen, which enhances ECM stiffness [208], thereby affecting the mechanosensory expression of cancer cells [209]. The interactions between cancer cells and CAFs are still more fascinating and novel, with studies indicating that cancer cells undergo alterations in YAP nuclear localization as a result of CAF capsules [210] and the CAF secretome [211]. In CAF capsules, CAFs exert compressive forces on cancer cells, indicating that it is not solely the stiffness of the ECM that can impact gene expression but also the forces imposed by fibroblasts present in the TME. Intriguingly, CAF capsules enveloping and exerting force on cancer cells have been found to suppress the growth of cancer cells [210], which may result in a jammed state. This indicates an intricate interplay between substrate/cell stiffness and gene expression in the act of triggering or stopping the movement of cancer cells. The interaction of cancer cells with other cells, such as immune cells, needs to be characterized in terms of jamming transition behavior. Similarly, the interaction of cancer cells with endothelial cells during the jamming transition should be investigated, as endothelial cells also possess mechanosensing and mechanotransductive mechanisms [3], which can in turn influence the behavior of cancer cells, such as the jamming transition. It remains unclear, however, how crosstalk between various cell types within the TME can directly cause unjamming transitions in the advancement of cancer cells. The development of new tools and technologies, such as long-term and highly resolved imaging, 3D cancer cell models, 3D-printed cell model systems, multiscale computational approaches, and the development of more comprehensive TMEs, will contribute to unraveling the molecular mechanisms underpinning the unjamming paradigm in cancer cell movement [212,213].

### 3.3. Temperature and Pressure Impact (Un)Jamming Transition Through the Control of Fluctuations

Cells in a collection are cooled to solidify them, which causes them to become solid but leaves them in a disordered arrangement. When cells are cooled, they experience a glass transition. The cooling procedure minimizes fluctuations that can be caused by temperature drops and/or pressure increases. All this results in a higher cell packing density. A key question concerns whether the jamming-to-unjamming transition involves thermal or non-thermal motion. Even in the face of gravity, corn on the cob can become stuck in the chute. Grains of sand in a heap can arrange themselves into a sloping, stable structure that is not at zero, which is referred to as the angle of repose. When the sand pile is adequately inclined, when someone hits the chute forcefully enough, the collective flow can start up again [214]. The systems discussed so far share the feature that a high enough perturbation stress exceeding the yield stress can reestablish flow. In the same way, cellular driving forces can exceed the local yield stresses to relieve a cellular collective, i.e., to unjam it [41].

The above are all examples where the energy hurdles far surpass the available thermal energy k_B_T, in which “k_B_” stands for Boltzmann’s constant and “T” denotes the thermodynamic temperature. In the absence of enough thermal energy to surmount energy hurdles, these systems are frequently characterized as “non-thermal” in nature. Similarly, as with each grain of sand in a sand pile being too bulky to be displaced by k_B_T and therefore being non-thermal, so every cell in a confluent tissue is non-thermal. Nonetheless, it may be helpful in some instances to envision larger-scale non-thermal mechanisms of local motion driven by the stochastic operation of molecular motors, propulsive forces, or some other factor that functions as an “effective temperature” [41,215,216]. A suitably strong random movement can locally unjam the cellular system and enable the cells to surmount energy obstacles and thus engage in exchange with their neighboring cells. The classification of systems as either thermal or non-thermal leads us to the topic of glass transition.

Fredberg has questioned the terminology of the material transition of a multicellular system as a “jamming transition”, as it appears to be more of a “glass transition”. This hypothesis seems to be reasonable. These transitions were only called “jamming transitions” for the sake of better memorability, without actually differing from the “glass transition” at a first glance, a phenomenon that was identified much earlier and has been thoroughly investigated [22,215,217,218]. The reason why the jamming transition is actually a glass transition is that when competition between energy barriers on the one hand and kinetic energy on the other hand determines the transition from liquid-like to solid-like behavior, such a transition is more accurately referred to as a “glass transition”. In a general sense, the motion can be external and non-thermal, as in the case of tapping on a culture dish of cells, or it can be internal and thermal, like the case of the glass transition of biopolymers [217]. Another non-thermal intrinsic motion is the driving force of a cell, which is produced by the coordinated action of molecular motors [22,28,127,219]. Contrary to the glass transition, the jamming transition, strictly speaking, does not rely on thermal or other motion. Instead, the jamming transition is solely a function of geometric features and thus constitutes a formal boundary of zero activity, that is, zero motion [218,220,221].

### 3.4. Control of Mechanosensitive Ion Channels and Transcription Factors in Unjamming Transitions

Apart from mechanical cues of the ECM scaffold, biochemical signals may play a role in the unjamming transition. This raises the following question: how could different biomechanical signals originating from the ECM control signal transduction during 3D cell migration? Cells are subjected to tensile stretch when moving though confined regions like narrow channels and densely packed ECM surroundings [222]. When the ECM is invaded and processes like intravasation and extravasation occur, migrating cells become squeezed because the pathways are narrowed [159,223]. In reaction to these physical constraints, cells exert forces to alter their shape and squeeze themselves through confined spaces, allowing them to migrate through restricted 3D regions. Jamming transitions during 3D-limited migration provide clues about the mechanisms by how cells can become stuck while migrating in the primary cancer or trying to penetrate into the ECM [27,43]. When cancer cells move through narrow passages within the tight ECM, they face elevated mechanical obstacles. Cell movement can be impeded if the mechanical forces applied by the cells exceed a specific threshold level, and the cells are unable to move or become jammed [169].

Mechanical signals have been demonstrated to remodel the junctions between epithelial cells via the elongation and contraction of the junctions by mechanosensitive channels [224]. The exact molecular mechanisms that convert mechanical signals into alterations in invasive behavioral patterns have not been fully elucidated. Previous studies have shown that exogenous expression of the mechanosensitive channel with high conductivity (MscL) in cancer cells prevents cells from entering confined 3D channels [223]. Piezo1 is another mechanosensitive channel of special importance. Piezo1 controls the proliferation and motility of cells in reaction to diverse mechanical inputs and modulates cell movement in both solitary and collective movements [225,226,227,228,229]. Significantly, Piezo1 is highly expressed in the majority of cancer types and is linked to poor survival outcomes [227,230,231]. During cancer cell movement, activating Piezo1 improves the capacity of cancer cells to travel when pressured and squeeze through tight spaces by causing a shift from F-actin-driven pseudopods over to bleb-facilitated cell motility [231,232]. The mechanical activation of Piezo1 resulted in augmented matrix breakdown, the development of actin protrusions, and an inflow of calcium ions [233,234]. Since Piezo1 channels are linked to the actin cytoskeleton via the cadherin-β-catenin compound [235], Piezo1 and E-cadherin could interact to pass on adhesion-cytoskeletal forces. An interesting idea is that Piezo1 mechanotransduction might be part of how E-cadherin expression is controlled because of how Piezo1 helps transfer mechanical forces that act on the plasma membrane from outside and inside the cell [227]. An intriguing study by Cai and colleagues has indicated that cell sorting and burst-like movement caused by the stiffness of the ECM are reliant on E-cadherin, which aids the activity of Piezo1 during the movement of cancer cells [171]. Consequently, the impact of mechanosensitive channels may also include control of E-cadherin turnover, thus connecting mechanotransduction to the dynamics of cell adhesion; it may perhaps serve as a mechanism for unjamming transitions. Thus, environmental cues seem to be linked to the unjamming transition. In addition, mechanosensitive transcription activators, YAP and TAZ, are involved in modifying the dynamics of cell adhesion processes and the expression of transcription factors that are highly expressed in various types of cancer [236]. YAP and TAZ have been the first genes found to be significant in the Hippo signaling route, which is a well-known signaling pathway involved in how organs form [237,238]. In terms of cell motility, YAP/TAZ have been shown to control the reorganization of the actin cytoskeleton and the maturation of focal adhesions, thereby promoting sustained motility [236,239,240]. Apart from biochemical signal transduction, there is compelling evidential support for the notion that regulatory mechanisms of YAP and TAZ are strongly impacted through mechanical signaling within cells [241].

In particular, the localized distribution of YAP alters when it is exposed to force [241,242,243]. It is assumed that YAP is activated and localizes in the nucleus when the nucleus is stretched, as occurs in cells moving on a rigid substrate [241,243]. In human breast tumors, YAP localization to the cell nucleus becomes more intense with increasing ECM stiffness, together with an up-regulation of pMLC, pFAK, and activated β1 integrin mechanosignal transduction [209]. It has recently been shown that epithelial cells grown on rigid substrates keep their rapid migration even when they move on softer substrates, and this “mechanical memory” relies on YAP [242]. The localized distribution of YAP in the cell nucleus is also assumed to be mainly due to the compression of the cell nucleus and not solely due to the stiffness of the substrate [244]. As cancer cells move through limited openings in stiff ECMs, the cells not only perceive stiffer substrates, but the cell nucleus also changes shape, for instance, by forming a nuclear piston [195], to make it easier to move through the barrier. The role of the cell nucleus needs to be explored more to see whether nuclear deformation is caused just by the substrate or is linked to other mechanisms that subsequently lengthen the nucleus, whereby a transition to an unjammed state is evoked.

## 4. Mechanochemical Cell–ECM Interactions Link EMT and Unjamming States in 3D Invasion

There is currently a vigorous debate whether the EMT and the unjamming transition of cancer cells are the same, separate, or interrelated. In many manuscripts, EMT and the unjamming transition are not clearly defined and appear to be used interchangeably without any consideration as to whether they refer to the same phenomenon or two separate ones. These two transitions could also influence one another and be impacted by the same factors. The subsections below first present the traditional role of EMT during the course of cancer. This is followed by an examination of whether EMT and unjamming transitions are separate, identical, or interlinked phenomena. Finally, the question of whether metastatic spread of cancer can take place in the absence of EMT but accompanied by a transition from jamming to unjamming is discussed, highlighting the fact that both transitions are separate from each other.

### 4.1. Classical Role of EMT in Cancer Progression

Everyone’s organ surfaces and hollow spaces are covered by a layer of cells that are all connected. Under homeostatic conditions, the epithelial collective stays firmly attached and settled. The epithelial collective turns liquid-like and moves around during morphogenesis, reorganization, or healing, and also when cancer spreads or metastasizes [245,246,247,248]. This shift from settled to migratory patterns has conventionally been interpreted as an expression of EMT or partial EMT (pEMT) (Table 2) [249,250,251,252]. In 1982, the EMT has been revealed; broadly explored, especially in the field of cancer progression; and precisely determined [250,253,254]. EMT is characterized through a gradual deterioration of the epithelial features, comprising impaired apical–basal polarity, disintegrated cell–cell connections, and compromised integrality of the epithelial cell layer and its associated barrier functionality. The progressive decline in epithelial characteristics is coupled with a gradual gain of mesenchymal features, like a stronger front–back polarity, the activation of EMT-initiating transcription factors, and the upregulated expression of the mesenchymal markers [255,256]. During this event, each epithelial cell has a propensity to detach from its adjacent neighbors, acquiring the ability to migrate and invade. It has been hypothesized that the epithelial–mesenchymal axis is bordered by clear epithelial and mesenchymal phenotypic extremes, which are distinguished by a progressive range of hybrid epithelial/mesenchymal (E/M) or partial (incomplete) pEMT phenotypes [249,252,257,258].

**Table 2:** Comparison between EMT and the jamming-to-unjamming transition (in cancer). Selected main molecular and physical features are listed for EMT and the jamming-to-unjamming transition.

Features	EMT	Jamming-to-Unjamming Transition
**Molecular features:**		
Alterations in gene expression	Downregulation of epithelial hallmarks like E-cadherin, claudins, and desmosomes, and upregulation of mesenchymal hallmarks like fibronectin, N-cadherin, and vimentin [246,259,260,261].	Downregulation of epithelial hallmarks like E-cadherin, and increased expression of N-cadherin. Vimentin causes **no** unjamming [203]; therefore, it can be hypothesized that E-cadherin expression and its localization are necessary for compression-induced unjamming transitions.
Regulation of transcription factors	A group of transcription factors, comprising two double zinc finger and homeodomain factors, such as ZEB1/2, the Snail family of zinc finger proteins (SNAI1/2/3), the family of bHLH factors (TWIST1/2, E12/E47), Krüppel-like factor 8 (KLF8), and Brachyury becomes active [262,263,264,265,266,267,268,269,270].	Activation of activator protein 1 (AP-1) transcription factors like JUN, JUNB, and ATF3 in unjamming [271].
Signal transduction routes	Oncogenic Ras and NF-κB signal transduction routes [272]. Several cellular signaling routes, such as TGF-β, Wnt/β-catenin, and the Hedgehog and Notch signaling routes, can trigger EMT, which often depends on the surrounding environment [273,274,275,276,277,278].	RAB5A triggers unjamming by promoting the internalization of the epidermal growth factor receptor (EGFR), leading to the hyperactivation of the extracellular signal–regulated kinase 1/2 (ERK1/2) and phosphorylation of the actin nucleator WAVE2 [17,271].
Reorganization of the cytoskeleton	Actin polymerization and intermediate filaments [279,280].	Actin remodeling promotes jamming-to-unjamming transition [271].
Perturbation of chromatin remodeling complexes	SWI/SNF, ISWI, CHD, and INO80 [281].	---
DNA methylome is subject to selective alterations in CpG methylation in specific genomic locations	Alterations in CpG methylation, such as E-cadherin promotor methylation and DNA methylation-linked silencing of miR-200 family members [282,283,284].	---
Increased coordination number	---	In the jamming phase, a hexagonal structure is preferable because it maximizes packing density and results in increased structural stiffness [18].
Non-affine deformations	It can be assumed that there is non-affine deformation [285].	It seems to be applicable to cells [286].
**Physical features:**		
Alterations in cell shape and polarity	Elongated cells with front–rear polarity [287,288].	Cell shape adaptions [41].
Attenuation of cell–cell adhesion/ disaggregation	Reduced cell–cell adhesion and individual cells [289].	No breakdown of cell–cell adhesions, barrier function, and polarity, and no expression of mesenchymal markers [62].
Resistance of anoikis	Reduces anoikis [290,291].	
Enhanced migration and invasion	Elevated migration as individual cells or as a collection of cells [289].	During the transition from jamming to unjamming, the cell layer migrates but keeps its complete epithelial nature [13,62].
Disordered state	Yes [292].	Yes [40].
Stiff-to flow transition of cancer cells	Yes [293].	In the jammed phase, each cell remains almost stationary, trapped by its immediate cell neighbors [294].
Discontinuity in coordination number	---	In analogy to soft matter, it is hypothesized to be increased in the unjamming phase [295].
Susceptibilities (first-order transition)	No.	Yes [41].
Critical density	No.	Yes [41].
Stiffness of the environment	Induces EMT [296].	Low ECM stiffness promotes the jamming-to-unjamming transition [36].
Compression	Facilitates of EMT [297].	Induces jamming-to-unjamming transition [32].
Contractility	Triggers EMT [298].	Promotes jamming-to-unjamming transition [294,299].
Viscoelasticity	ECM viscoelasticity controls TGFβ1-driven EMT [300].	Drives jamming-to-unjamming transition [301].

This 1D range of states can certainly be considered an oversimplification [249,302], but there is broad consensus that pEMT enables cell movement without full mesenchymal specialization [303,304,305,306]. In pEMT, cells communicate with their neighbors by keeping some connection integrity, losing part of their apical–basal polarity, and gaining a graded front-back polarity and ability to move [249,252]. In addition, EMT/pEMT has been implicated in the cells of highly aggressively growing tumors, confers stem cell-like characteristics and increased resistance to cytotoxic cancer medications on cancer cells, and it may be necessary for the fibrotic reaction [307,308]. Cells subjected to EMT exert apical–basal force by the assembly of apical–basal myosin II structures, which strongly suggests a mechanical impact on tissue remodeling, such as in cancer initiation and progression [309]. In line with developmental events [308,310,311,312], wound healing processes [313,314], and tissue fibrosis [315], EMT/pEMT is a generally agreed upon idea for explaining collective migration in cancer [103,316,317,318,319], which is why EMT is seen as necessary in a lot of cases [146,246,303,308,314,320].

### 4.2. Are EMT and Jamming-to-Unjamming Transitions the Same, Separate, or Interlinked Phenomena?

The key role of EMT in the movement of cancer cells, invasiveness, and metastasis is well documented [321]. Ilina and coworkers have seen different types of collective migration in unjammed cancer cell groups that display varying EMT status after E-cadherin downregulation [27]. In particular, by adjusting the cell–cell adhesion strength, various liquid phases have been found in triple-negative 4T1 breast cancer cells that migrate together along a 2D collagen-glass boundary. Highly migratory and elongated cancer cells can actually move with a high level of coordinated movement in the face of strong cell adhesion, which represents an active nematic phase, or with a minimal degree of coordination in the face of weak cell adhesion, which represents an active liquid phase [27]. In contrast to Ilina and coworkers, Kang and coworkers reported that triple-negative MDA-MB-231 breast cancer cells that collectively infiltrate high-density collagen exhibit a consistent radial tendency toward decreased cell volumes, more regular morphology, and slower migration [33], which leads to the hypothesis that these cells are more closely related to a jammed state. The slower kinetics of the invasive ramifications that form are characterized by decreased tangential speed and coordinated radial movements among adjacent cells at the invasive perimeter [33]. This apparently paradoxical finding of a collective invasion caused by jamming is in line with the idea of a wandering “fixed herd” [80], where the cells are locked in a fixed state with no local reorganization, but they can move in a collectively and directed manner because their ability to move sideways is blocked. The collective invasion into mesenchymal cells may be due to the formation of supracellular actin cable networks [63], which may raise surface tension at the boundaries and enable coordinated movements within ECM with higher density. Kang and coworkers could not detect this kind of actin structure within MDA-MB-231 spheroids [33], so other mechanisms, like cell–ECM signal transduction and cell contractility [322], probably carry out an important function. Overall, the results suggest that both jamming and unjamming greatly contribute to tissue fluidity and its collective invasion characteristics. That is why limiting the ECM, along with a linear route to progressive tissue liquefaction through unjamming [17,27], can reestablish orderly migratory invasion through progressive re-jamming and encourage cooperation between nearby cells with weak adhesion. The precise mechanisms are still unclear, but Kang and coworkers’ results show that jamming and unjamming transitions are a much more complex phenomenon than the conventional view, highlighting the usefulness of a unifying interpretative framework based on a jamming phase diagram. Beyond that, however, the question of how much the unjamming transition is independent of EMT or partial EMT, related to it, or even required for it is still up in the air [323].

The EMT did not appear to be essential for initiating the migratory dynamics and invasion of cancer cells in the past. This realization came as a surprise, although, in retrospect, it is probably not that surprising. Based on a resting, polarized epithelial state, EMT and unjamming transition are currently considered alternative routes to cell migratory processes. When the morphological and molecular obstacles necessary for EMT are substantial, it is much easier for unjamming to occur, as these requirements are quite low. A transition from a jammed phase to an unjammed phase can be achieved by a slight but meaningful change in location on the jamming phase diagram with the 1/cell density, 1/cell adhesion, and cell motility on the three axes, whereby, for instance, an epithelial phenotype is maintained throughout. The maintenance of an epithelial phenotype before and after the transition from a jammed to an unjammed state and vice versa is the main difference to EMT, where epithelial phenotype transitions to a mesenchymal phenotype and vice versa need to occur. In EMT, the change in tissue phenotype is inevitable and therefore both transitions appeared to be fundamentally different [299].

Numerous essential biological functions, like tissue repair, demand tightly arranged cell monolayers/tissues to transition from a jammed solid condition to an unjammed liquid condition. Despite the fact that quantitative investigations assume that alterations in cell shape in isolation can result in unjamming, there has been no conclusive experimental evidence to validate this assumption, since liquefaction as a consequence of density changes has not been entirely excluded in living systems. On top of that, the idea that a cell can adjust how it can move adds to the challenge, because even in arrangements of stiff active particles, any sort of difference in how they move on their own can have a significant impact on the overall dynamics. Arora and coworkers have therefore developed and constructed a monolayer of synthetic cell imitations and investigated their collective performance. Through systematic prolongation of the self-propulsion duration, the researchers have identified a cell shape-controlled, density-independent, reentrant jamming phase transition [99]. In particular, they have reported that cell shape and shape diversity are mutually restrictive in the confluent boundary region and exhibit the same universal scaling behavior seen in confluent epithelia. Dynamic heterogeneities, nevertheless, did not follow the same scaling, with rapid cells exhibiting suppressed morphological plasticity, which, according to the simulations, is due to a temporary restriction imposed on these cells through their slower neighboring cells [99]. In their experiments, Arora and colleagues clearly demonstrated a morphodynamic relationship and showed that geometric limitations alone can determine the jamming/unjamming of epithelia [99]. In addition to this traditional method of unjamming, cells, in contrast to inert particles, can also undergo deformation to overcome the restrictive properties of compaction and promote fluidization of the system [72,73,294]. The competitive actions of cell contractility and cell–cell adhesion in the Vertex model of confluent epithelia [53,324] result in a density-independent but cell shape-dependent unjamming transition [40]. When jammed, the cells take on a clearer hexagonal form, while in a liquid state, they become more of an elongated shape.

**Figure 4:**
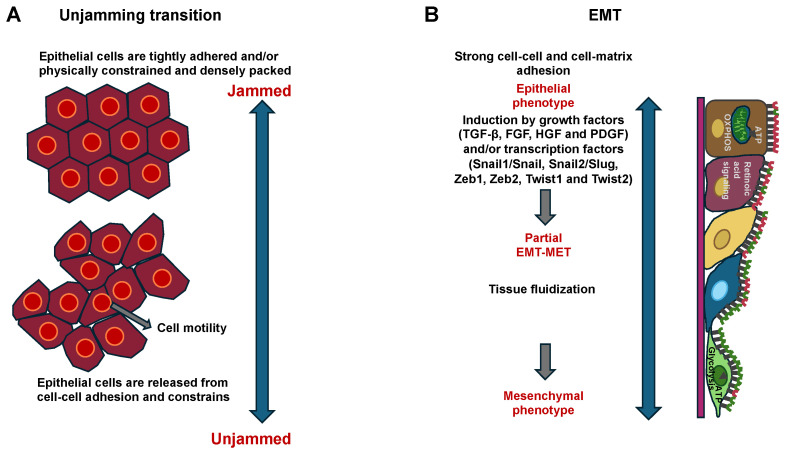
Schematic drawing of the unjamming transition and EMT models for the collective movement of cancer cells. There is still a lot of discussion on whether these two transitions are the same, distinct, or interrelated. (**A**) Schematic of the transition between jammed and unjammed states in confluent epithelial cell layers. In the jammed state, the cells are non-motile, whereas in the unjammed state the cells become motile. (**B**) Schematic representation of the EMT model in collective cell movement. FGF = fibroblast growth factor; HGF = hepatic growth factor; PDGF = platelet derived growth factor; TGF-β = transforming growth factor- β; and Zeb = zinc finger E-box binding (Zeb) homeobox family proteins.

The unjamming is regarded as a motility induction event triggered by various sources like chemoattractants, electrical currents, or the development of wounds [294]. In conditions where cells are enclosed and tightly crowded, like in solid cancers, they are capable of migrating in various forms in the face of physical forces imposed on them through the tissue microenvironment [5,67,161,171,177,325,326,327,328]. The switch from a fixed to a mobile condition has mainly been studied using the EMT model, where epithelial cells, that is, solid tissue, change their cell polarity and cell–cell adherence and become invasive, leading to tissue liquefaction (Figure 4) [8,249,252,326].

Epithelial migration is marked by tight cell–cell interactions, such as elevated E-cadherin and decreased vimentin expression levels and focal adhesions, whereas mesenchymal migration is characterized by low cell–cell interactions, such as decreased E-cadherin, elevated N-cadherin, and elevated vimentin expression and reduced focal adhesions in the following cells [249,329]. The application of compressive stress to a single layer of primary human bronchial epithelial cells has led to the stimulation of a transition from a jammed to an unjammed state, which is not primarily driven through EMT [13,62]. This points to a possible difference in the characteristics in the unjamming transition. Epithelial and mesenchymal cells exhibit opposite collective behavioral patterns. When tightly packed, epithelial cells do not have the energy to counteract high bonding and tensile forces (Figure 4A) [8,13,17]. Unjamming is a phenomenon that can arise when tension or restriction is removed and cell adhesion is strong, leading to a firmly connected group in which all cells participate in collective movement in an equal manner (Figure 4A). In contrast, mesenchymal cells move as a loosely bound group, like the collective movement of animals, bacteria, and self-propelled particles (Figure 4B) [330]. From a biological perspective, the EMT process is the traditional theory explaining how epithelial cells attain a migratory trait [256]. In the EMT, cell–cell adhesion becomes less tight, and epithelial adhesion markers like E-cadherin are switched out for N-cadherin (Table 2). Leader cells retain strong focal adhesions, whereas follow-up cells exhibit attenuated adhesions and decreased traction forces [252]. Conversely, unjammed bronchial epithelial cells that move collectively retain tight cell–cell connections and simultaneously display fluid-like behavior marked through longitudinal cell shapes [62]. Consequently, EMT and unjamming transition programs within the airway epithelium of the lungs have come to be recognized as separate phenomena [62], even though no studies have been conducted on other epithelial systems in this context, and this differentiation is potentially not as straightforward. More broadly, it remains unclear to what extent EMT and unjamming transition contribute separately, sequentially, or jointly to morphogenesis, growth, and tissue reorganization. The question of whether the lifting of blockades (unjamming) in various settings is controlled by a conserved group of triggering signals, transcription regulators, and downstream effectors is still elusive [15,17,271,331]. In the airway epithelium, when subjected to mechanical compression resembling bronchospasm, the subsequent unjamming transition is conveyed through a sequence of processes that enhance actin polymerization by the recruitment of integrin–ECM adhesion complexes and enhance cell motility by AP-1 transcription factor activation downstream of ERK and JNK signaling routes (Table 2) [271,331]. Collectively, these results indicate that the unjamming program is not the consequence of a solitary signaling route, but it instead involves a concerted action of downstream signaling routes that participate in development, cellular fate selection, energy metabolism, cytoskeletal reorganization, and adhesive interplay with the ECM.

It is common knowledge that the unjamming transition can occur without EMT. This allows cells that maintain a strictly epithelial phenotype to unjam themselves and migrate collectively and efficiently without impairing the protective barrier function [62]. The opposite claim that EMT can occur without interference from the unjamming transition remains unproven. When a small proportion of cells in an epithelial layer carry out partial EMT, the individual cells can produce driving forces that enable them to move within the monolayer or even out of it. In vitro, it has been demonstrated that adding a growing amount of mesenchymal cells to epithelial cells leads to an enhancement of collective movement [332]. It is unclear, however, whether this type of cells, which can be seen as mesenchymal activators, can interfere with the rest of the epithelial cells, thereby breaking down the jamming state of the layer and evoking the cell collective to start migrating. Intriguingly, signs of both cell jamming and unjamming have been found in the mesenchyme of the branching airways of developing chicks [26]. Thus, it can be questioned whether the jamming and unjamming states are only present in epithelial cells. Although there are differences between the unjamming transition and the EMT, the question arises as to whether there is a connection between these two transitions nevertheless, which will be discussed below.

### 4.3. Is There a Linkage Between EMT and Unjamming Transition?

The plasticity of cancer cells enables them to alternate between different modes of migration, allowing them to invade the ECM and thus enhancing the likelihood of metastasis. In the field of mechanobiology and biophysics, the term “unjamming” is applied to different dynamic phenomena in biological systems. The observed migration patterning varies from collective movement of layers or clusters [15,17,22,28,62] to single-cell squashing occurrences [63,94] or T1-transitions [333], whereby other transitions such as T2 and T3 are generally excluded from typical collective movement analyses. These distinct migration modes can be identified through the characteristic speed correlation length, migratory persistence, or packing density of the collaborative migratory cell groups. Cells alter their polarization and migration orientation in response to interactions with neighboring cells [38,334], which can result in enhanced collective motility. Theoretical modeling considers this by introducing an additional alignment interacting term. The resultant phase diagram of the jamming includes four different phase states as follows: solid/jammed, solid flock/flowing solids/active nematics, liquid flock/flowing liquids, and liquid [15,80]. These states provide a more detailed representation of the experimentally identified migration modes and demonstrate that “unjamming” can refer to several different types of movement. This brings up the idea that the dynamic signatures of cells in motion might be a clue about how strong the interactions between cells are. The migration modes seen in cells are also similar to those found in active nematic liquids. Self-propelled rods that exhibit a higher aspect ratio, like elongated mesenchymal cells, show phases of flocking, lane formation, and isotropic orientation with sharply defined phase transitions. In addition, although the flocculation movement in active nematics is collective, it is internally jammed at the local level [335], which can also be found in living tissues [16,63]. The fact that cellular systems can also exhibit mixed states of jamming and unjamming should also be taken into account, as can be observed, for instance, in spheroids incorporated into collagen, where the center may remain jammed while the outer envelope has begun to liquefy [17,336].

There is still broad consensus that EMT and unjamming transitions represent two separate routes by which cancer cells acquire invasive properties. The extent to which these routes are interlinked remains uncertain. It is even discussed whether these two transitions are really distinct from one another. The following section describes how Van der Net and colleagues investigated this question by conducting 3D spheroid invasion assortments of lung adenocarcinoma A549 (epithelial) and melanoma MV3 (mesenchymal-like) cancer cell lines in collagen hydrogels. They modified the invasive nature of the cells either by adding transforming growth factor (TGF)-β to encourage EMT or by blocking cell-mediated matrix breakdown with matrix metalloproteinase (MMP) inhibitors, which also made the matrix structure more porous [35]. By performing quantitative image analysis to trace spheroid invasion, Van der Net and coworkers observed that the start of invasion primarily relied on the porosity of the matrix. In addition, the ability to invade has been shown to be linked to vimentin levels, whereas the rate at which spheroids spread was mainly influenced by metalloproteinase MMP1 levels and therefore depended on cell–matrix interplay. Morphological analysis demonstrated that spheroids in hydrogels with small pores exhibited solid-like (non-invasive) behavior. In contrast, in hydrogels with large pores, the cells were mesenchymal-like, as they have transitioned to a liquid-like (strand-based cells) or gas-like (spreading cells) state. These results are in line with the unjamming transitions predicted in current models of cancer invasion as a function of cell motility and matrix restriction, but they reveal that cell motility and matrix restriction are connected through EMT-driven matrix breakdown [35].

Traditionally, the unjamming transition and EMT have been considered separate processes, but it is still open to question whether they can be combined in certain situations. EMT is no longer viewed as a solitary, black-and-white process, but it rather embraces a range of intersecting biological features. RNA and protein profiling analyses indicate that cells can assume intermediate stages or pass through a partial EMT, which represents a phenomenon that is more accurately termed epithelial–mesenchymal plasticity (EMP) [256,337]. EMT refers to the capacity of cells to easily transition between epithelial and mesenchymal modes of behavior, which is predominantly controlled by EMT transcription factors (EMT-TFs), such as Snail1, Snail2, Zeb1, Zeb2, and Twist (Table 2). It is noteworthy to note that EMT-TFs are also found in non-epithelial cell types, which complicates the conventional perception of EMT [256]. Intriguingly, it has been demonstrated that cancer stemness, cell survival, metabolic alterations, and cancer drug resistance can emerge from EMT-TFs [256,338]. Moreover, the lack of the metastatic suppressor gene NME1 in epithelial cancer cells leads to a hybrid intermediate cell phenotype because of changed cadherin expression [339] and decreased surface tension [340]. These parameters linked to EMT-TFs and genes are additionally encouraged in settings with high matrix stiffness during the course of jamming-to-unjamming transitions.

Cai and coworkers investigated multicellular sorting and migration under restriction. They showed that partial EMT, which is marked by low E-cadherin expression and high vimentin expression, combined with decreased matrix stiffness, can lead to the explosive (burst-like) movement of human breast cancer cells and non-cancerous cells [171]. This burst-like migration appears solely after the removal of the restriction, suggesting that EMP and the unjamming transition may be linked under certain circumstances. Another recent investigation similarly found that cancer cell spheroids in hydrogels containing small pores display solid-like characteristics, whereas those in hydrogels with large pores tend to behave in a gas-like manner [35]. In addition, Van der Net and colleagues showed that EMT and unjamming are connected to the activity of MMPs, supporting the breakdown of the ECM and improving cell movement. More studies on how EMT and unjamming transitions are related are needed. Therefore, it is recommended that a leader cell enveloped by solid tissue at the site of the primary solid tumor be stimulated to transition from a solid, jammed phase to a liquid, migratory state [18,43]. The unjamming transition out of the blockade is controlled not merely by intercellular communications within the solid tumor, but also through interactions between the cells and the ECM scaffold [160,341].

### 4.4. Can Metastatic Spread Occur Without EMT but with the Jamming-to-Unjamming Transition?

In carcinomas, the majority of metastases can currently be traced back to circulating multicellular tumor cell clusters (CTCs), which often keep an epithelial appearance over the entire course of metastasis [44,342,343]. Cheung and Ewald also hypothesized that maintaining an epithelial phenotype may be necessary for CTCs to successfully progress from the primary solid tumor to remote metastatic target locations [344]. When cells maintain an epithelial phenotype during all steps of the metastatic cascade, the concept that EMT constitutes the sole or most significant route to cell motility is not valid, as in EMT, the migratory cells display a mesenchymal phenotype. Nevertheless, assuming that the process is not EMT, what alternative physical mechanism could trigger and persist migration? Since cell jamming has been described in in vivo murine cancer models [43], a simple but unproven hypothesis cannot be ruled out, that is, that this process, which promotes the spread of metastatic cancer, could be the transition from jamming to unjamming. There are at least three supporting observations that support this hypothesis. The first observation is that at the primary tumor site, the movement of a leading cell and its followers starts when these cells switch from a solid-like jammed state to a liquid-like unjammed state. The second observation is that when they approach the bloodstream, they form a CTC and are subject to destabilizing mechanical influences from the shear forces transmitted by the blood. The CTC responds by stabilizing and reverting to a solid-like jammed phase. The third observation is that when these CTC cells arrive at their destination, for instance, the metastatic niche, they either enter a dormant (jammed) state [345], or start to invade the surrounding tissue. In the latter case, they invade the surrounding tissue and form a secondary tumor. The concept that metastasis necessitates EMT and its reverse process, MET, thus contrasts with the opposing hypothesis that metastasis entails comparatively less extreme events, such as the transitions between solid (jammed) and liquid (unjammed) phases of the cell layer, in which robust collectivity and an epithelial phenotype remain intact during the entire process. An interesting but counterintuitive conclusion emerges with respect to possible approaches to modifying these processes is that enhanced intercellular adhesion facilitates unjamming and vice versa. In addition, the impact of intercellular friction and contact impediment of movement must be investigated in more depth in a jamming phase diagram [14,97].

## 5. Discussion on the Importance of Jamming and Unjamming

In many manuscripts, the jamming-to-unjamming transition is hypothesized to serve as a universal mechanism for the collective migration of cancer cells during malignant progression of cancer. This appears to be an oversimplification of the published experimental findings. Hence, it is questionable whether this hypothesis will hold true, as until now, only a few cancer types, such as breast, cervix, lung, kidney, and skin (melanoma) cancers, have been explored. Conventional cancer cell lines were mainly used as cellular model systems. These were cultivated with varying amounts of bovine serum and then compared with each other, which does not necessarily seem appropriate. In most of these experimental approaches, the effect of the ECM scaffold and other nearby or matrix-embedded cells [2,3] is largely ignored. This also applies to spheroid culture models, in which other cells, immune cells, stromal cells, and endothelial cells were ignored for the sake of simplicity. Moreover, the oxygen content within the spheroid was not investigated, so it is ultimately questionable whether these model systems still represent the latest state of the art.

From a mechanobiological perspective, it is crucial to control the behavior of biological systems over long distances and periods of time so that conclusions can be drawn about general mechanisms. There are a bunch of processes that can be involved in this regulation, like genetic networks, biochemical systems, or mechanical systems. These active interventions are powerful, but they need energy and constant feedback. In contrast, jamming has been identified as a fundamental phenomenon in a wide range of systems, including biological, chemical, and physical systems. In both inert and living systems, a slight variation in a relevant control factor, like the volume fraction, can cause a huge shift in material characteristics even without extensive structural alterations. Cells that form a multicellular tissue are governed by some similar physical restrictions, while also being active and capable of responding. This is why it is quite difficult to apply physical principles to them. It can therefore be challenging to identify the suitable parameters for controlling jamming within a multicellular ensemble. It is nevertheless evident that jamming contributes to defining cell behavior and tissue micromechanics; however, the question arises as to its general applicability to, for example, all cancer types and all stages of malignant tumor progression, i.e., all steps in the metastatic cascade. Within several development systems, it has been revealed that overcrowding and the connected alterations in cell jamming can cause layer separation and, occasionally, differentiation. Since the control mechanisms are identical throughout the tissue, the cells react to nearby cues while staying in line with more distant neighbors. Interestingly, a comparable innate coordinative behavior is evident when cells are eliminated from the layer as follows: the force equilibrium realigns the dividing cells to mitigate the tension caused by the loss of cells and to preserve the integrity of the layer [346,347]. It could be that the jammed cell layer within a developing tissue controls its reaction, at least in part, in a globally coordinated manner. Its response is based solely on local signals from the environment and attempts to minimize tension within the layer, either by ejecting cells when overcrowded or by aligning dividing cells to optimally plug voids within the layer.

Cell jamming also permits the coordinative behavior of cells at the tissue level, which appears to be a feature common to both healthy and diseased states, such as cancer. A key fact is that most of the systems investigated are all near the jamming transition. Since the jamming transition enables major mechanical alterations with comparatively minor modifications of state parameters like viscosity and cell–cell adhesion force, the cells gain remarkable mechanical regulation of their response to the microenvironment [348]. Conversely, functioning near a jamming transition point can also entail challenges. For instance, continued differentiation and delamination in mature tissue because of fluctuations in cell tension are unwanted and could impair tissue performance.

Energy metabolism naturally plays a pivotal part in cancer, but data linking energy metabolism to jamming and unjamming is still scarce, particularly in the field of cancer research. There are some data available from wound healing research. For instance, in the confluent monolayer of MDCKII cells, increasing migration capacity, fluidity, and unjamming of the anterior margin zone are associated with a decreasing redox ratio in the cytoplasm, a shorter NADH lifetime, and an increase in mitochondrial membrane potential and glucose uptake [349]. In this scheme, unjamming leads to a switch in energy metabolism away from oxidative phosphorylation to glycolysis, which is more rapid but lower in efficiency with respect to ATP output per glucose molecule. The unjamming-induced switching to glycolysis is evocative of the Warburg effect observed in cancer cells. Therefore, it can be hypothesized that the same results could also apply to cancer cell layers, which would demonstrate the importance of the jamming-to-unjamming transition.

Are cell jamming and unjamming phases essential for the development of cancer and its malignant progression? The answer to this question is currently uncertain due to the limited data available. In particular, many studies fail to take into account the environment surrounding cancers and its influence on jamming–unjamming behavior. Nonetheless, it can be assumed that the jammed phase of multicellular tissue is also compatible with tumors subject to pressure, which encourages an energetically economical and mechanically metastable resting state. The unjammed phase, conversely, corresponds to an essential but energetically costly adaptation of multicellular tissue, such as cancerous tissue to disturbances. This adaptation necessitates reactions, such as collective cell migration, liquefaction, or reorganization, as observed during the malignant progression of cancer. Thus, it seems likely that cell jamming and unjamming are necessary for the metastatic spread of cancer, possibly even as a universal phenomenon. Moreover, the transition from jamming to unjamming appears to be independent of the well-known and universal EMT, suggesting that it may even play an equally important role in the malignant progression of cancer. As the data available here are not yet extensive, this remains to be seen. Therefore, much experimental work remains to be performed, such as analyzing the transition from jamming to unjamming in 3D TMEs over time using live cell imaging to determine whether and where the transition from jamming to unjamming and the reverse phenomenon occur in cancer. The 3D force mapping analysis over time can aid in figuring out the role of forces in the jamming-to-unjamming transition. Moreover, the heterogeneity of the TME, such as embedded stromal and immune cells, could be addressed as well.

## 6. Conclusions and Future Directions

Due to the heterogeneity of the TME, cancer cell migration is an intricate event comprising multimodal migration, which poses challenges for the therapeutic management of metastatic cancer. There are different cell types inside the cancer, which accumulate due to the mechanical crosstalk between the cells, like cell adherence and stiffness. In addition, cancer cells engage with the encompassing ECM scaffold, where the stiffness and viscoelasticity of the ECM can impact gene expression and movement. Along with the clear biochemical paths of cancer cell invasion, the jamming transition model is proposed. Thereby, the mechanical characteristics of the cells propel the sorting within the cancer and generate regions with jammed, solid-like cell mass that are encircled by unjammed, fluid-like cell migration. Unjammed, fluid-like cells are present at the cancer periphery with improved accessibility to the ECM, where they are activated by the ECM and rearrange it to facilitate more efficient cancer cell migration and, subsequently, invasion. Future research needs to keep figuring out the molecular mechanisms of the unjamming transition in analogy to EMT to separate the various biochemical and mechanical signaling routes in the migration of cancer cells. Apart from straightforward 2D approaches to describe jamming-to-unjamming transition, there needs to be more emphasis on determining a 3D shape index for the rigidity transition, as it has been started in 2018 by Merkel and Manning [113]. It may be combined with a novel cell polarity and cell shape analysis approach, which is termed Polarity-jam in 3D cell systems [350], whereby, together with cell polarity, junction and cell morphology and organelles like nuclei–Golgi polarity can be determined. In addition to analyzing the cells, the cellular environment must be reconstructed and characterized at the same time in a dynamic manner and on different length scales so that its mechanical and biochemical effects on the transition from jamming to unjamming and vice versa can be investigated in a functional manner. Finally, understanding the jamming-to-unjamming transition seems to be relevant for future drug development in various cancer types.

## Figures and Tables

**Table 1:** Jamming–unjamming transition in certain cancer types and certain normal healthy tissue types. The strengths and limitations are listed.

Cancer or Tissue Type	Description/Reference	Strengths	Limitations
Breast cancer	Altered expression of cell–cell adhesion like E-cadherin and β-catenin molecules MCF-7 and 4T1 (derived from the mammary gland tissue of a mouse BALB/c strain) cell lines [27]	Identification of cell density control via 3D tissue junctions for physical guidance of collective movements independent of cell–cell junction composition and stability. Downregulation of E-cadherin and p120-catenin led to a transition from coordinated to uncoordinated collective motility across the extracellular junction.	Experimental analysis is largely based on a 2D cross-sectional analysis utilizing 2D trajectories. For tissue sections, the analysis has been carried out using 3D trajectories for cell movements. Fixated tumor specimens have been analyzed in serial section slices. The cell spheroids are rather simple as they contain only the cell line.
Breast cancer	Adhesive differences in MCF-7 and MCF-10A [31]	Migrating epithelial (cancer) cells represent an active, non-equilibrium system, and the cell monolayer exhibits glass-like behavior, implying jamming action as the basis for intercellular interactions. Phase contrast time lapse microscopy has been utilized.	EMT and the jamming-to-unjamming transition are not regarded as separate events. Two epithelial cell lines have been employed that largely differ in cell adhesion. The migratory capacity of the cells has been analyzed on rather stiff flat culture dishes in 2D.
Breast cancer	MCF10A, MCF10A.Vector; MCF10A.14-3-3ζ; MCF10.ErbB2, MCF10AT; and MCF10CA1a [28]	The concept of jamming has been shown to be important for cancer cell lines with different invasion capabilities. Higher velocities were linked to larger cooperative cell clusters across diverse cell lines. Structure and migration dynamics were consistent with previous theoretical descriptions of the cell jamming. Jamming-associated migratory mechanisms have been detected.	These model systems obviously lack a number of factors that play a role in vivo, in particular immune cells, cancer stem cells, connective tissue, vascularity, and 3D.
Breast tissue	MCF-10A with RAB5A expression [15]	RAB5A can trigger large-scale, coordinated movements across dozens of cells and ballistic movements in monolayers that are otherwise kinetically locked. This is related to elevated traction forces and the elongation of cell protrusions, which orient themselves to the local speed. A simple model based on mechanical connection tension and an active mechanism for cell realignment for the speed of self-propelled cells identifies monolayer dynamics modes that account for the onset of motion through a mixture of large-scale directed migration and local unjamming.	2D monolayers and cell lines.
Breast tissue	MCF-10A and MCF10DCIS.com cells (ductal carcinoma in situ (DCIS) cell line that has been derived from a xenograft lesion following two trocar passages of the premalignant cell line MCF-10AT) [17]	Investigation of unjamming in a number of normal and tumorigenic epithelial 2D and 3D collectives. Unjamming in tumor spheroids is linked to persistent and coordinated rotations that gradually reshape the ECM while fluidizing the cells at the peripheral region. The endogenous ERK1/2 signaling path (RAB5A) is a physical–chemical trigger that initiates the collective invasion and spread of otherwise jammed cancers. Spheroid surrounding ECM is explored.	Tumor slices have utilized that are only 2D. Only a few cell lines are explored.
Breast tissue	MCF-10A and 4T1 with E-cadherin knock down [32]	Long-term mechanical compression leads to cell arrest in benign epithelial cells and increases the migration of cancer cells in transitions that correlate with cell shape. These findings prompted them to examine the roles of cell–cell adhesion and substrate traction in unjamming transitions. Cadherin-driven cell–cell adhesion controls reaction to compressive stress and drives of unjamming in stressed monolayers. Compression stress cannot trigger EMT in unjammed cells. Traction force microscopy revealed the reduction in traction forces in compressed cells within the monolayer independent of cell type and movement.	Only a few cell lines are explored in a 2D setting.
Breast tissue	MCF-10A cell spheroids exhibit predominantly epithelial traits and MDA-MB-231 cell spheroids expresses primarily mesenchymal traits [33]	Non-equilibrium phase separation based upon jamming and unjamming transitions seem to offer a unifying physical concept for cellular migratory dynamics inside and out of a tumor.	MDA-MB-231 spheroids have been generated only by addition of 2.5% Matrigel. The comparison of MCF-10A and MDA-MB-231 spheroids is hampered by the different amounts of serum and serum types, such as 5% horse serum for MCF-10A and 10% fetal calf serum for MDA-MB-231.
Breast and cervix tissue	Tumor explants obtained from patients with two types of carcinomas (four breast carcinomas and twelve cervical carcinomas) are examined for their nuclear shape in cell clusters [34]	Tracking viable cells in explants of solid tumors from patients demonstrates that an elongated cell and nucleus shape and low nuclear density characterize the unjammed phase. Cancer cell unjamming represents an emergent physical feature that promotes cancer progression.	2D histological analyses of the shape of cells and cell nuclei; only two different cancer types (cell lines) have been explored.
Bronchial epithelial	Maturation and strengthening of cell–cell and cell–matrix adhesions in immortalized human bronchial epithelial cells (HBECs) [14]	Complex dynamics in the aging of a cell monolayer, in which cell movement becomes gradually slower over time, while the distance over which cell movements are correlated first rises and then diminishes. Alteration of this behavior is independent of cell density but relies on the ripening of cell–cell and cell–substrate adhesions.	2D monolayer. Two cell types are explored.
Kidney tissue	Madin–Darby Canine Kidney (MDCK) [29]	Traction forces that propel collective cell migration are generated primarily many cell rows away from the leading edge and extend over vast distances.	A canine cell line has been employed. 2D traction force measurements.
Lung and skin tissue	3D spheroid invasion analysis of two distinct human cancer cell lines, such as the highly metastatic and mesenchymal-like MV3 melanoma cells and epithelial A549 lung carcinoma cells [35]	3D spheroid invasion assay using in collagen-based gels. The timing of invasion correlated with matrix porosity and vimentin levels, whereas spheroid expansion rate linked with MMP1 levels. Cell motility and matrix restriction are linked via EMT-related matrix breakdown.	Only a few cell lines are explored. The analysis of unjamming transitions must be performed instead of considering different states.
Skin tissue	MV3 melanoma cells (mesenchymal-like cells) [36]	3D interface assay with a gap between two high-density collagen grids has been employed to jointly analyze cancer cell invasion efficacy, invasion mode and MMP dependence. Inhibition of collagen breakdown severely impaired migration in 3D collagen in a density-dependent fashion, but migration controlled by the interface remained effective and took place through cell jamming.	The ECM consists only of collagen. Only based on one cell line.
Skin tissue	Human A431 epidermoid carcinoma cells migrate collectively in confined microchannels. Most separation events, referred to as “ruptures,” concern individual A431 cells that detach, but ruptures of large groups of about 20 cells within wider channels can also be seen [37]	Phase field cell motility model has been created by defining three different cell states, such as follower, guided, and highly motile “leader” cells, depending on their spatial location. Ruptures of about 20 cells within wider channels can also be observed.	Unjamming is required but not sufficient to induce ruptures.

## Data Availability

No new data were created or analyzed for this study. Data sharing is not applicable to this article.

## Abbreviations

The following abbreviations are used in this manuscript:

AFM	Atomic force microscope
ALI	Air–liquid interface
AR	Aspect ratio
CAFs	Cancer-associated fibroblasts
DCM	Deformable cell model
ECM	Extracellular matrix
EMT	Epithelial to mesenchymal transition
HBECs	Human bronchial epithelial cells
MCT	Mode coupling theory
MDCK	Madin–Darby canine kidney
PCP	Planar cell polarity
pEMT	Partial epithelial to mesenchymal transition
pMLC	Phosphorylated myosin light chain
SPP	Self-propelled particle
SPV	Self-propelled Voronoi
TME	Tumor microenvironment
